# Rules Governing Selective Protein Carbonylation

**DOI:** 10.1371/journal.pone.0007269

**Published:** 2009-10-05

**Authors:** Etienne Maisonneuve, Adrien Ducret, Pierre Khoueiry, Sabrina Lignon, Sonia Longhi, Emmanuel Talla, Sam Dukan

**Affiliations:** 1 Laboratoire de Chimie Bactérienne – Aix Marseille Université - UPR 9043-CNRS, 31, Chemin Joseph Aiguier, Marseille, France; 2 Service de micro séquençage et de spectrométrie de masse – CNRS-31, Chemin Joseph Aiguier, Marseille, France; 3 Architecture et Fonction des Macromolécules Biologiques, Aix Marseille Université - CNRS, UMR 6098, Marseille, France; Griffith University, Australia

## Abstract

**Background:**

Carbonyl derivatives are mainly formed by direct metal-catalysed oxidation (MCO) attacks on the amino-acid side chains of proline, arginine, lysine and threonine residues. For reasons unknown, only some proteins are prone to carbonylation.

**Methodology/Principal Findings:**

We used mass spectrometry analysis to identify carbonylated sites in: BSA that had undergone *in vitro* MCO, and 23 carbonylated proteins in *Escherichia coli.* The presence of a carbonylated site rendered the neighbouring carbonylatable site more prone to carbonylation. Most carbonylated sites were present within hot spots of carbonylation. These observations led us to suggest rules for identifying sites more prone to carbonylation. We used these rules to design an *in silico* model (available at http://www.lcb.cnrs-mrs.fr/CSPD/), allowing an effective and accurate prediction of sites and of proteins more prone to carbonylation in the *E. coli* proteome.

**Conclusions/Significance:**

We observed that proteins evolve to either selectively maintain or lose predicted hot spots of carbonylation depending on their biological function. As our predictive model also allows efficient detection of carbonylated proteins in *Bacillus subtilis*, we believe that our model may be extended to direct MCO attacks in all organisms.

## Introduction

Oxidative damage by reactive oxygen species (ROS) is associated with ageing [1], [2] and several neurodegenerative diseases [3]–[5]. Indeed, ROS are generated as by-products of cellular metabolism and have the potential to induce significant biological damage. There is ample evidence to support the notion that the most important mechanism of protein oxidative damage is metal-catalysed oxidation (MCO), resulting in cleavage of polypeptide backbone, cross linking, and modification of the amino acid side chains leading to the loss of protein function and to structural alteration [6]. Among the various oxidative lesions, protein carbonylation is extensively used to monitor oxidative damage due to its irreversible and irreparable nature, and is used in the development of sensitive immunochemical methods that detect oxidative damage [2], [7]. Carbonyl derivatives are essentially formed by direct MCO attacks on the carbonylatable amino-acid side chains of arginine (R), lysine (K), threonine (T) and proline (P) residues. Carbonyl derivatives of lysine, cysteine and histidine are also formed by adduction of reactive aldehydes derived from the MCO of polyunsaturated fatty acids. Carbonyl derivatives of lysine residues can be formed by secondary reactions with reactive carbonyl compounds on carbohydrates and advanced glycation/lipoxidation end products [8].

Approximately 10% of the proteome is more prone to carbonylation during ageing, starvation or disease [2], [7], [9], [10]. There are several possible explanations for this specificity, including (i) the presence of a transition metal in the protein (MCO is an intrinsic problem for protein containing transition metals) or (ii) the localisation of these proteins close to ROS generating sites, but the molecular basis for the apparent specificity of protein carbonylation remains unclear [11]. Recently, several groups worldwide have been working on the identification of carbonylated sites (CS) within proteins from various organisms [12]–[16]. These studies have led to two major observations: (i) sites are selectively carbonylated among all carbonylatable sites, and (ii) CS are mainly located at the protein surface. Although these studies have contributed to our understanding of protein carbonylation specificity, we are still unable to predict sites more prone to carbonylation or to understand the propensity of proteins to undergo carbonylation.

We used MALDI-TOF and LC nano-ESI MS/MS to identify CS in oxidised bovine serum albumin (BSA) after *in vitro* MCO, as well as in 23 *in vivo* carbonylated proteins (CP) from *Escherichia coli*, chosen as a model organism. The identification of CS led to the concept of carbonylation hot spots (HSC). We then derived some rules for predicting HSC and thereby of proteins more prone to carbonylation. Finally, we developed an *in silico* model (web tool available at http://www.lcb.cnrs-mrs.fr/CSPD/) allowing an efficient and accurate prediction of sites and proteins more prone to carbonylation in the *E. coli* proteome, and more generally for predicting CP generated via direct MCO attacks.

## Results

### BSA carbonylation content as a function of the MCO level

**Figure 1:**
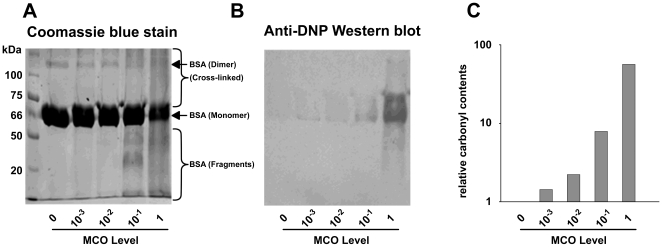
BSA oxidation state as a function of the MCO level. BSA was reacted at increasing MCO levels and analysed by (A) SDS/PAGE (10% polyacrylamide) with Coomassie staining or (B) after DNPH-derivatisation and blotted onto PVDF followed by carbonyl immunostaining. (C) The relative carbonyl content of the BSA monomer using Quantity One densitometry analysis (Biorad) analysis. The carbonyl content of the MCO untreated sample was set to 1.

We first addressed whether some carbonylatable sites within BSA were more prone to carbonylation than others. Thus, we set up a range of MCO levels leading to an increase in the BSA carbonyl content. We observed a decrease in the content of BSA monomer and dimer, paralleled by an increase in fragmentation and the amount of cross-linked products after BSA was treated with increasing MCO levels (Fig. 1A). MALDI-TOF analysis confirmed the presence of fragments, monomers and dimers within the BSA sample after MCO treatment (supplementary Fig. S1). Concomitantly to these phenomena was an MCO-dependant increase in the carbonyl content of BSA monomers (Fig. 1B). The relative carbonyl content gradually increases with increasing MCO levels, being 2-fold to 60-fold higher than in untreated BSA (Fig. 1C). Taken together, these observations indicate that the best MCO range for our study was comprised between 0.01 (where carbonyl content starts to increase) and 1 (where the amount of BSA monomer significantly decreases).

### Identification of carbonylated sites in BSA

We next used LC nano-ESI MS/MS and MALDI-TOF to identify CS in the BSA samples obtained after treatment at the various MCO levels, as described in the Materials and Methods section.

#### Carbonylation is highly selective and most CS are solvent accessible

**Figure 2:**
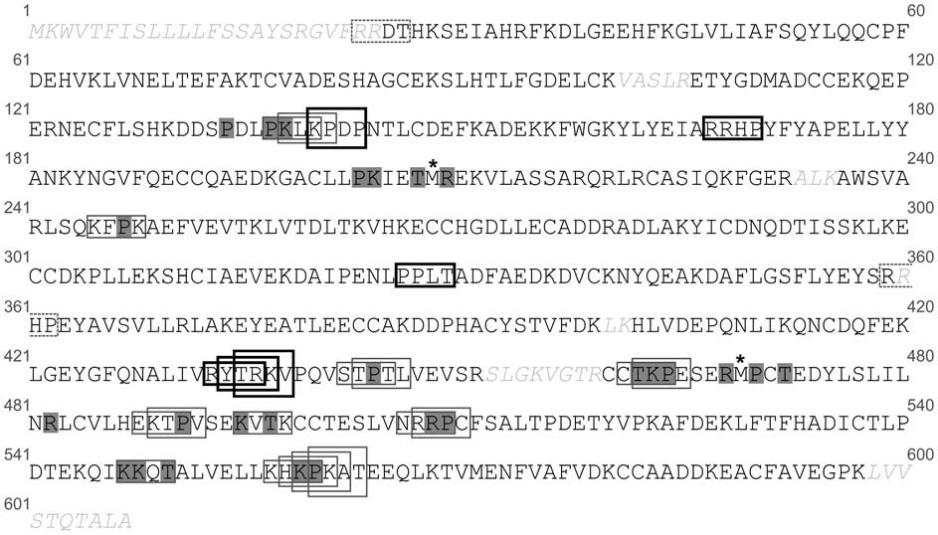
Distribution of BSA site-specific oxidation. BSA is a secreted protein and its precursor contains 607 amino acids (Accession number P02769), whereas the mature form lacks the first 24 residues and contains 583 amino acids. This figure displays the amino acid sequence of the BSA precursor with CS identified by mass spectrometry shaded in gray and oxidised methionines shown by an asterisk (*). Amino acids shown by gray italic characters correspond to regions not found by mass spectrometry analysis (less than 10% of the primary sequence). RKPT-enriched regions (3 carbonylatable sites within a 4 amino acid sequence window) are represented within framed boxes. Thin boxes correspond to RKPT-enriched regions containing at least one CS, whereas thick boxes correspond to RKPT-enriched regions in which no CS were identified. Boxes represented by a dotted line correspond to RKPT-enriched regions in which no CS were identified, but which contain residues that were not covered by mass spectrometry analysis of tryptic peptides.

BSA is a polypeptide comprised of 607 amino acids containing 143 (23.5%) carbonylatable sites (R, K, P, or T). The identified tryptic BSA peptides cover more than 90% of the BSA protein sequence (Fig. 2). Within these peptides, 133 sites are carbonylatable (R (20), K (55), P (28) or T (30)) and 86% of them are solvent accessible, judged based on the visual inspection of the crystal structure of human serum albumin (PDB ID: 2I30), whose sequence shares 75% of identity with that of BSA. We identified 3, 14 and 26 CS out of a total of 126 carbonylatable sites, at 0.01, 0.1 and 1 MCO levels, respectively (Table 1), where the 3 and 14 sites observed at the lowest MCO levels (i.e. 0.01 and 0.1) were also observed at the highest MCO level (i.e. 1). These results clearly indicate that carbonylation is highly selective and that most CS are solvent accessible (Table 1). Proline appears to be the most reactive of the carbonylatable residues (Table 1).

**Table 1:** Identification of specific BSA carbonylated sites.

	MCO Levels		
Identified CS	0.01	0.1	1
P_464_, P_470_	*	*	*
K_559_	*	*	*
P_203_, ^▒^P_247_, P_491_, P_509_, P_560_		*	*
K_204_, K_495_, K_548_		*	*
R_468_, ^▒^R_508_		*	*
T_550_		*	*
^▒^P_134_, P_137_, P_444_			*
K_138_, K_463_, K_547_			*
R_209_, R_482_			*
T_207_, T_462_, ^▒^T_472_, T_497_			*
Number of solvent accessible CS	3	12	22
Total number of CS	3	14	26
Percentage of CS	2.3%	10.5%	19.6%

CS as a function of the MCO level. The asterisks indicate the MCO levels where CS were experimentally found.^(▒)^ CS found to be buried as judged by visual inspection of the crystal structure of the homologous human serum albumin (PDB ID: 2I30).

#### BSA CS are mainly present in RKPT-enriched regions

Interestingly, 75% of CS were clustered at the highest MCO level, and were separated in some cases by as little as one residue (Fig. 2). This prompted us to test whether CS were mainly located within RKPT-enriched regions. An RKPT-enriched region is defined by the presence of at least 3 carbonylatable sites within a 4 amino acid sequence window, resulting from an optimal combination of specificity and sensitivity (supplementary Table S1). Thus, 25 RKPT-enriched regions were identified within the BSA sequence using these criteria (Fig. 2). Carbonylation analysis indicated that carbonylatable sites located within RKPT-enriched regions are approximately 4 times more prone to carbonylation than those external to these regions. Indeed, within the identified tryptic peptides, 38.6% (17 of 44) of carbonylatable sites within RKPT-enriched regions were carbonylated, whereas only 10.1% (9 of 89) of the carbonylatable sites external to these RKPT-enriched regions were experimentally found to be carbonylated. However, carbonylatable sites from 6 RKPT-enriched regions were not shown to be carbonylated (Fig. 2), thus suggesting that other rules are required for specific carbonylation of these regions.

### Propagation of carbonylation renders RKPT-enriched regions more prone to carbonylation

#### Mass spectrometry analysis reveals an order in the propagation of carbonylation

At the lowest MCO level, two of the three CS are located within the R_459_-L_483_ region that contains two RKPT-enriched regions (Fig. 2 and Table 1), indicating that this is the most reactive region. We identified 3 to 5 peptides that form this region using LC nano-ESI MS/MS: these peptides contain 0 to 7 oxidised sites depending on the MCO level, (Table 2 and supplementary Fig. S2). BSA MCO-dependent oxidation heterogeneity was also observed when MALDI-TOF analysis was performed (supplementary Table S2). Moreover, at the highest MCO level used, the presence of peptides identified at different oxidative stages suggests a reaction pathway with several steps of successive oxidation resulting in the carbonylation of all carbonylatable sites present in this area. Based on these observations, we suggest that the reaction pathway is initiated by carbonylation of two accessible sites, P_464_ and P_470_, followed by the oxidation of M_469_, then by the carbonylation of R_468_ and finally by the carbonylation of T_462_, K_463_, T_472_, R_482_. Interestingly, the buried T_472_ site was not carbonylated unless sites P_464_, R_468_ M_469_ and P_470_, which are solvent accessible, were already carbonylated. Similar observations were found at the lowest MCO level, but not all carbonylatable sites in this area were carbonylated (Table 2). All these results indicate a specific order in the propagation of carbonylation.

**Table 2:** Mass Spectrometry analysis reveals a reactional pathway of carbonylation.

	MCO Levels		
peptides	1	0.1	0.01
R_(459)_.CCTKPESERMPCTEDYLSLILNR.L_(483)_	x		
K_(463)_.PESERMPCTEDYLSLILNR.L_(483)_	x	x	
K_(463)_.PESERMPCTEDYLSLILNR.L_(483)_	x	x	x
K_(463)_.PESERMPCTEDYLSLILNR.L_(483)_	x	x	x
K_(463)_.PESERMPCTEDYLSLILNR.L_(483)_	x	x	x

BSA peptides encompassing the most reactive region (R_459_-L_483_) as a function of the MCO level. CS and oxidised methionine are shaded in gray and asterisks show the MCO level where the peptides were identified.

#### Carbonylatable site reactivity to oxidation depends on the presence of a neighbouring carbonylated site

The results described above suggest that oxidation reactivity of a carbonylatable site depends on the presence of a neighbouring CS. To validate this hypothesis and to determine whether it could be extended to all carbonylatable sites, we investigated the location of all CS within the peptides identified by LC nano-ESI MS/MS. If the MCO level was increased from 0.1 to 1, 8 of the 12 sites found to be newly carbonylated were located in close proximity to a previously CS (Fig. 2 and Table 1). These newly CS could not be detected without the prerequisite presence of a site already carbonylated within each identified peptide (data not shown). Interestingly, three of the four other sites specifically carbonylated at the MCO level of 1 are prolines, known to be the most reactive of residues.

Thus, the presence of a CS increases the reactivity of a neighbouring carbonylatable site and may partially explain why carbonylatable sites within RKPT-enriched regions are more prone to carbonylation.

### Analysis of carbonylated proteins in *E. coli*

We next addressed whether the observations obtained *in vitro* with BSA could be generally applied to proteins known to be carbonylated *in vivo* via direct MCO attacks. For this purpose, we used *E. coli*, as carbonylated proteins in *E. coli* are only generated in *vivo* via direct MCO attacks. Using protein extracts from cells harvested in the exponential phase of growth and 2D-electrophoresis analysis as previously described [17], we detected 40 carbonylated spots, representing roughly 10% of the total number of protein spots (supplementary Fig. S3). We identified 23 CP [17]. Interestingly, these CP show a percentage of carbonylatable residues (R, K, P, and T) (20.75%+/− 1.6) that is close to the value obtained for the complete *E. coli* proteome (19.95%+/− 3.7), indicating that CP were not carbonylated due to an enrichment in carbonylatable sites.

#### Carbonylation is highly selective and most CS are solvent accessible

Overall, we identified 83 CS (out of 1585 carbonylatable sites present within the sequences covered) within the 23 *E. coli* CP (Table 3 and supplementary Table S3). Similar to our findings during the *in vitro* BSA study, proline appears to be the most reactive site (supplementary Table S3). The fact that only 5% of the carbonylatable sites were carbonylated once again highlights a strong selectivity during carbonylation.

**Table 3:** CS found within the set of 23 *E. coli* proteins.

				Location of CS			
Gene name	Accession number	PDB	Identified carbonylated peptides	Solvent accessible	Disordered region	Buried	Unknown
*aceE*	NP_414656.1	2G28		^(1)^P_787_			
*acnB*	NP_414660.1	1L5J	R_(735)_.LWAPPTRMDAAQLTEEGYYSVFGK.S_(761)_ K_(373)_.DVAESDRGFSLAQKMVGR.A_(392)_	^(1)^K_387_^(1)^R_391_^(2)^P_489_		^(2)^P_739_^(2)^P_740_	
*atpD*	NP_418188.1	2JDI (E-F)	K_(70)_.DLEHPIEVPVGK.A_(83)_	^(2)^P_75_^(3)^P_79_^(2)^P_115_			
*clpB*	NP_417083.1	1QVR		^*(1)^R_418_			
*dnaK*	NP_414555.1	1DKG (D)	K_(414)_.NTTIPTKHSHSCQVFSTAEDNQSAVTIHVLQGER.K_(446)_ K_(55)_.RQAVTNPQNTLFAIK.R_(71)_	^(1)^R_56_^(1)^T_60_^(2)^P_90_	T_416_ T_417_ P_419_ P_420_		
*eno*	NP_417259.1	2FYM (A-D)	K_(201)_.GMNTAVGDEGGYAPNLGSNAEALAVIAEAVK.A_(233)_	^(2)^T_204_^(2)^P_214_			
*fabH*	NP_415609.1	2GYO (A,B)		^(2)^P_47_			
*fhuA*	NP_414692.1	1BY5 (A)		^(2)^P_96_			
*fusA*	NP_417799.1	1KTV (A,B)	K_(605)_.KAKPVLLEPIMK.V_(619)_	^*(3)^T_348_^(3)^P_524_^(2)^P_570_^(2)^K_609_^*(2)^T_681_		^(3)^P_615_	
*groL*	NP_418567.1	2EU1 (A-N)	K_(468)_.GGDGNYGYNAATEEYGNMIDMGILDPTKVTR.S_(502)_ K_(28)_.VTLGPKGRNVVLDK.S_(43)_	^(2)^T_30_^(2)^P_3_^(2)^K_34_^(1)^P_496_^(1)^T_497_^(1)^T_500_^(1)^R_501_^(1)^K_498_		^(2)^R_36_, ^(1)^P_113_, ^(1)^P_235_	
*htpG*	NP_415006.1	2IOP (A-D)	R_(531)_.LTDTPAIVSTDADEMSTQMAK.L_(553)_	^(2)^T_535_^(2)^P_536_^(2)^T_541_		^(1)^R_33_	
*imp*	NP_414596.1	1KV9 (A)					R_650_
*leuS*	NP_415175.1	2V0G (A, D)		^(1)^T_695_			
*ompA*	NP_415477.1	2GE4 (A)	R_(190)_.FGQGEAAPVVAPAPAPAPEVQTKHFTLK.S_(219)_		P_198_ P_204_ P_206_ P_208_ T_212_ K_213_ T_216_ T_127_		
*pnp*	NP_417633.3	1E3P		^*(2)^P_544_			
*proS*	NP_414736.1	2J3M (A,B)		^*(2)^P_340_			
*purA*	NP_418598.1	1KKF (A)	K_(401)_.RIEELTGVPDIISTGPDRTETMILRDPFDA.-_(432)_	^(2)^P_410_^(2)^P_418_^(2)^R_420_^(2)^T_421_^(3)^T_423_		^(2)^T_416_, ^(2)^P_429_	
*rpoB*	NP_418414.1	2PPB (C, M)	R_(976)_.AVLVAGGVEAEKLDKLPR.D_(995)_	^*(1)^R_1301_			K_203_ P_993_ R_994_
*^▒^rpsA*	NP_415431.1	1EFU (B, D)					T_270_
*sucC*	NP_415256.1	1JKJ (B, E)	R_(225)_.QPDLREMR.D_(234)_	^(1)^P_227_^(1)^R_230_			
*Tsf*	NP_414712.1			^(2)^T_270_			
*tufB*	NP_417798.1	2FX3 (A)	K_(295)_.PGTIKPHTKFESEVYILSK.D_(315)_ K_(314)_.DEGGRHTPFFK._(326)_ R_(205)_.AIDKPFLLPIEDVFSISGR.G_(225)_ R_(373)_.FAIREGGRTVGAGVVAKVLS.-_(386)_	^(2)^P_210_^(3)^P_214_^(2)^P_296_^(2)^T_298_^(3)^P_301_^(3)^T_303_^(2)^R_319_^(2)^T_321_^(2)^P_322_^(2)^P_353_^(3)^T_383_^(3)^K_391_		^(3)^P_366_	
*^▒^yaeT*	NP_414719.1				P_326_		
total				55	13	9	6

The table lists the carbonylated peptides containing at least two CS. Whenever possible, assessment of the structural environment and of solvent exposure of isolated CS was done by visual inspection of the relevant pdb or homologue file, thus allowing location of CS within ^(1)^ alpha helices, ^(2)^ loops, or ^(3)^ beta strands. The ^(*)^ symbol highlights CS that are not conserved in the corresponding homologous PDB file and are replaced by a non-carbonylatable residue. In those cases where structural data were either completely ^▒^(NP_415431.1, NP_414719.1) or partially lacking (NP_414555.1, NP_417799.1, NP_414596.1, NP_415477.1 and NP_418414.1), we carried out structural predictions to assess whether the CS occurred in ordered or in disordered regions (see Materials and Methods section).

We then assessed whether carbonylation occurs in solvent accessible sites; thus, depending on whether structural data were available or not, we analysed or predicted the structural environment of each CS. We analysed the structural environment of 64 CS based on existing structural data. Among them, only 9 were buried, whereas 55 were found to occur within solvent accessible sites, clearly indicating that CS are predominantly found within solvent accessible sites. Among all of the CS occurring in regions of known structure (64), 36 were located within loops, 17 were located within α-helices, and 11 were located within β-strands (see Table 3 and supplementary Table S3).

For those cases for which no structural data were available, we used disorder predictions to assess whether the CS were located in ordered or disordered regions. Among the 19 CS for which structural data were lacking, 13 were located in a short disordered region (<30 residues), whereas the remaining 6 CS were located in regions that were previously predicted to be structured (see Table 3 and supplementary Table S3). No reliable prediction could be made on solvent accessibility for all the sites whose structural environment was predicted, as all of these sites were located within short disordered regions. Indeed, Liu and co-workers noticed that long regions (>70 residues) of non regular secondary structure (NORS) are generally solvent accessible [18], but short regions of disorder (<30 residues) may correspond to loops connecting regular secondary structure elements.

Overall, these data indicate that CS are preferentially located within solvent accessible sites and are not associated with a preferential structural environment.

#### CS are preferentially detected in RKPT-enriched regions

Finally, 12 of the 23 identified CP contained more than 2 sites of carbonylation, yielding a total of 72 CS (Table 3). Around 50% of these CS were clustered, separated by as little as one residue (Table 3). These results indicated that carbonylatable sites located within RKPT-enriched regions are approximately 4 times more prone to carbonylation than those external to these regions. Indeed, among the identified tryptic peptides, 11.3% (36 out of 317) of the carbonylatable sites located within RKPT-enriched regions were carbonylated, whereas only 3.5% (47 out of 1268) of carbonylatable sites external to these RKPT-enriched regions were experimentally found to be carbonylated.

Thus, *in vitro* (BSA) and *in vivo* (*E. coli*) studies indicate that RKPT-enriched regions are involved in selective protein carbonylation if CP are generated via direct MCO attacks.

### A predictive model for the identification of sites and proteins more prone to carbonylation

To identify sites and proteins more prone to carbonylation via direct MCO attacks, we developped a predictive carbonylated site and protein detection model (CSPD model).

#### Rules leading to the detection of predicted carbonylation hot spots (HSC) in the E. coli proteome

**Figure 3:**
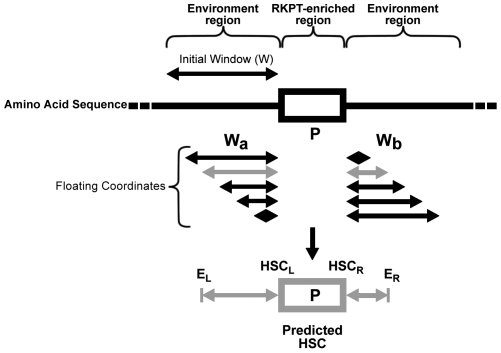
Schematic model of the detection of predicted HSC. The details of the principle of detection are described in Materials and Methods section. A predicted HSC (grey box) is defined by two regions. A) An RKPT-enriched region (3 carbonylatable residues within a sequence of 4 amino acids (R, K, P, T; 3; 4) containing at least one proline (P; 1; 0). B) A specific environment around an RKPT-enriched region, enriched in various residues: (i) iron binding sites (D, E, Y, H, C, namely 1 residue within a window of 2 residues (D, E, Y, H, C; 1; 2) and 8 residues within a window of 29 residues (D, E, Y, H, C; 8; 29); (ii) hydrophobic amino acids (A, V, G, I, namely 1 residue within a window of 2 (A, V, G, I; 1; 2)); (G namely 2 residues within a window of 14 (G; 2; 14)); and (iii) (P, T) with 2 residues occurring within a window of 21 residues (P, T; 2; 21). E, environment; r, right; l, left and w, window.

As indicated above, the presence of a local enrichment of RKPT is not sufficient to lead to effective carbonylation. Sequence analysis of the proximal RKPT-enriched regions, which were experimentally determined to be carbonylated in *E. coli*, allowed us to define additional rules for the prediction of sites and proteins more prone to carbonylation. Accordingly, a predicted HSC is defined as an RKPT-enriched region containing at least one proline, with a specific environment enriched in (i) iron binding sites (D, E, Y, H, C), and in (ii) hydrophobic amino acids (A, V, G, I) (all rules are indicated in Fig. 3), and assume to be more prone to carbonylation. Therefore, a protein containing a predicted HSC is defined as a predicted CP.

#### Predictive CSPD model allows effective detection of HSC and CP in the E. coli proteome

Parameters used when applying these rules were optimised so as to allow a maximal discrimination between the set of 23 CP [17] and the proteome of *E. coli*. Under these conditions, 19 of the 23 CP of the set (82.6%) contained at least one predicted HSC, whereas around 4 times less proteins from the *E. coli* proteome have a predicted HSC (21.2%). We then addressed whether carbonylatable sites within predicted HSC are indeed more prone to carbonylation than carbonylatable sites occurring external to these predicted HSC. Among the set of 23 CP, 4 of the 12 predicted HSC present within the coverage sequences obtained by mass spectrometry analysis were experimentally found to be carbonylated (supplementary Fig. S4). Twelve of the 58 carbonylatable sites within the 12 predicted HSC (20.7%) were carbonylated. By contrast, only 71 CS out of a total of 1540 carbonylatable sites external to HSC (4.7%) were carbonylated, thus indicating that carbonylatable sites within predicted HSC are five times more prone to effective carbonylation suggesting that HSC are indeed more prone to carbonylation. We used various protein sets to test the performance of the predictive CSPD model: a set of *E. coli* proteins composed of (i) 40 previously identified CP [7], [19] and (ii) 29 proteins identified in this study as not carbonylated (in healthy exponential cells), as judged by immunodetection and mass spectrometry analysis (supplementary Table S4). The performance of the predictive CSPD model was evaluated by jackknife testing, which yielded a sensitivity of 73%, a specificity of 75% and a predictive positive value (precision) of 81%. These results support the ability of the predictive CSPD model to predict with a good efficiency sites and proteins more prone to carbonylation via direct MCO attack in *E. coli*.

#### Efficiency of the predictive CSPD model in organisms other than E. coli

We also checked whether the predictive CSPD model also identified CP formed via direct MCO attacks in other organisms. For this purpose, we analysed three sets of experimentally identified CP from *Bacillus subtilis*, *Saccharomyces cerevisiae* and *Arabidopsis thaliana* [20]–[22]. *B. subtilis* CP which are only formed via direct MCO attacks, were detected with a good efficiency indicating that the predictive CSPD model can also detect CP generated via direct MCO attacks in other organisms (Table 4). For those organisms in which carbonyl derivatives are formed not only via direct MCO attacks, but also via secondary reactions, the predictive CSPD model was not useful, even though the percentage of predicted CP found in the set of proteins experimentally shown to be carbonylated was higher than that obtained for the complete proteome.

**Table 4:** Percentage of predicted CP in three organisms.

	*B. subtilis*	*S. cerevisiae*	*A. taliana*
***CP***	*71.3%*	*40%*	*51.1%*
***Proteome***	*20.2%*	*26.7%*	*29.6%*

The predictive CSPD model was run on both the set of experimentally detected CP and the total proteome of *B. subtilis* [21], *S. cerevisiae* [22] and *A. thaliana* [20].

### Analysis of *E. coli* predicted carbonylated proteins

#### Predicted HSC and HSC environments contain unique sequences within the E. coli proteome

Next, we further characterised predicted CP in the *E. coli* proteome. The CSPD model allowed the detection of 1253 predicted HSC within 899 predicted CP, whereas the remaining 3364 other proteins of the *E. coli* proteome were predicted to possess no HSC. Predicted CP contain up to 7 HSC, with most of them harbouring only 1 HSC (72.3% = 650/899) or 2 HSC (19.2% = 174/899) (data not shown). Finally, more than 60.8% (762/1253) of HSC have a specific motif for an RKPT-enriched region. Predicted HSC are selected based on an enrichment of specific amino-acid types within their proximal environment (supplementary Fig. S5). Thus, we checked whether a consensus could be identified for some amino-acid positions in the HSC environment (see Materials and Methods section). No consensus could be identified for any position within the HSC environment (supplementary Fig. S5). Taken together, these results clearly indicate that the HSC environment is typified by a number of features, rather than by the conservation of specific residues at key positions.

#### Protein shuffling analysis reveals that in predicted CP the presence of HSC is independent from amino acid composition

**Figure 4:**
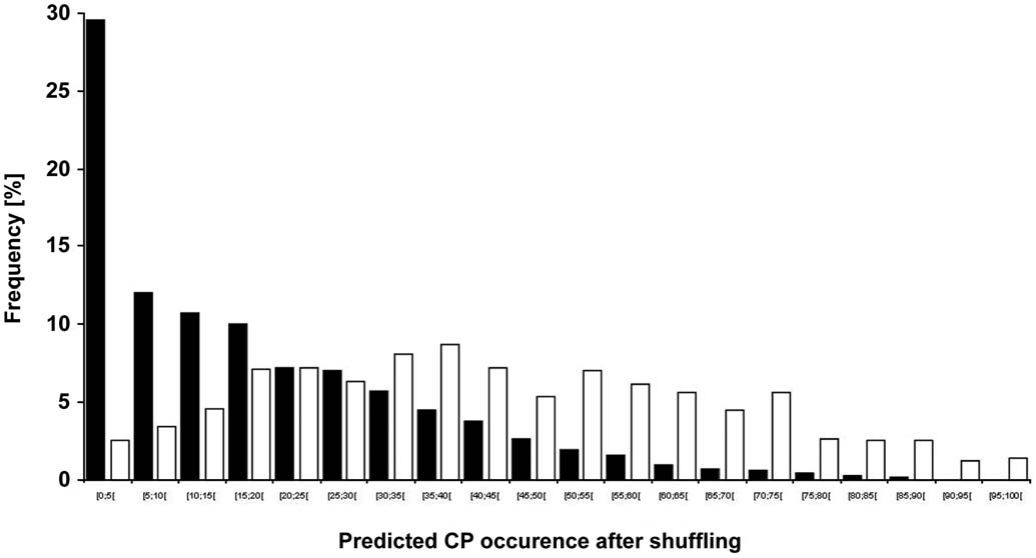
Effect of amino acid sequence shuffling of E. coli proteins on predicted HSC occurrence. We estimated the predicted CP occurrence for each protein of the *E. coli* proteome after shuffling (see text). A frequency distribution of the obtained values was then generated and plotted either for the set of proteins predicted to be carbonylated (899 proteins, white bars) or for the set of proteins predicted to be non-carbonylated (3364 proteins, black bars) in the *E. coli* proteome.

Next, we checked whether the HSC occurrence within predicted carbonylated and non-carbonylated sets of proteins arises from their amino acid compositions. Thus, we calculated the percentages of amino acids or amino acid groups involved in rules governing the predictive CSPD model, for both sets of proteins (i.e. predicted CP and non-CP). No significant differences were observed between the set of predicted CP and that of proteins predicted to be non-carbonylated (Table 5). This observation indicates that the occurrence of HSC is not dependent on an enrichment in carbonylatable residues alone, nor does it only arise from an enrichment in amino acid types typifying the environment of HSC. These relative percentages could be due to the cumulative effect of the overall set of 899 (predicted CP) or 3364 proteins (predicted non-CP). We therefore performed a similar analysis to test whether the occurrence of HSC within each protein was due to amino acid composition or due to the primary structure (amino acid position). To check the importance of the amino acid composition and position for each individual protein, we shuffled the *E. coli* proteome, such that for each *E. coli* protein, 1000 shuffled protein sequences were generated (see Materials and Methods section). For each set of 1000 shuffled protein sequences arising from a given *E. coli* protein, we estimated the percentage of shuffled proteins possessing at least one HSC using the predictive CSPD model (predicted CP occurrence after shuffling). We then calculated this percentage for all sets of shuffled protein sequences, where all these sets were obtained by shuffling either the 899 predicted CP, or the 3364 proteins predicted to be non-carbonylated. In this way, for each protein of the *E. coli* proteome, we estimated how often a predicted CP occurred after shuffling. A frequency distribution plot was generated by sorting the proteins in each set (i.e. predicted CP and non-CP) as a function of their predicted CP occurrence after shuffling (Fig. 4). For the family of 3364 proteins predicted as non-carbonylated, the frequency of distribution decreases with increasing percentages of predicted CP occurrence after shuffling (see black bars in Fig. 4). Thus, for this latter set, the persistence of relatively few predicted CP even after shuffling clearly indicates that it is the amino acid composition of these proteins that is responsible for the scarcity of predicted CP. Conversely, for the set of predicted CP, no clear relationship could be observed between the frequency distribution and the shuffled HSC occurence (see white bars in Fig. 4). We reasoned that if the amino acid composition were the only parameter responsible for the presence of predicted HSC in these proteins, then we would observe, for predicted CP (white bars), a mirror effect of results obtained with predicted non-CP (black bars). The absence of such a mirror effect establishes that the primary structure also plays a role in determining the presence of HSC in these proteins.

**Table 5:** Percentages of amino acids and amino acid groups in the *E. coli* proteome and in the sets of predicted carbonylated and non-carbonylated proteins.

	%P	%G	%RKPT	%PT	%AVGI	%DEYHC
*E. coli* proteome	4.3+/−1.7	7.14+/−2.4	19.95+/−3.7	9.67+/−2.5	29.74+/−5.5	17.28+/−4.6
Predicted CP	4.95+/−1.5	7.48+/−1.9	20.85+/−2.6	10.42+/−2.0	28.97+/−4.1	19.13+/−2.9
Predicted not CP	4.14+/−1.7	7.05+/−2.6	19.71+/−4.0	9.47+/−2.6	29.95+/−5.7	16.78+/−4.8

Mean percentages of amino acids or amino acid groups involved in rules governing the predictive CSPD model were calculated for the entire proteome (4243 proteins), the set of predicted CP (899 proteins) and the set of proteins predicted to be non-carbonylated (non-CP, 3364 proteins). Standard deviations from the mean are also shown.

#### Highly expressed proteins are enriched in predicted CP

**Figure 5:**
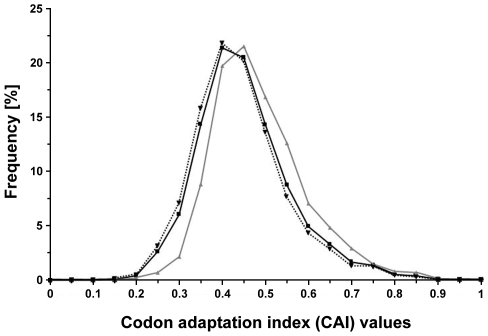
Analysis of the expression of genes encoding predicted CP. Frequency distribution of CAI (codon adaptation index) values, within the entire proteome of *E. coli* (4243 proteins, dark line), predicted CP (899 proteins, gray line) and predicted non-CP (3344 proteins, dotted line). The 95% confidence intervals of the mean were [0.4474–0.4538], [0.4776–0.4915], and [0.4379–0.4451] for the entire proteome, for carbonylated and non-carbonylated proteins, respectively.

Next, we addressed whether a relationship exists between protein abundance and the presence of predicted CP. Therefore, the codon adaptation index (CAI) was calculated for each *E. coli* protein, and frequency distribution of CAI values were analysed, as a function of the presence of predicted CP (Fig. 5). We found that higher values of CAI (linked to protein abundance) are statistically associated with predicted CP, as the 95% confidence intervals of the average CAI values are [0.4776–0.4915] for predicted CP and [0.4379–0.4451] for proteins predicted as non-CP. These results indicate that predicted CP are linked to highly expressed genes.

#### The abundance of predicted CP depends on the functional protein category

**Figure 6:**
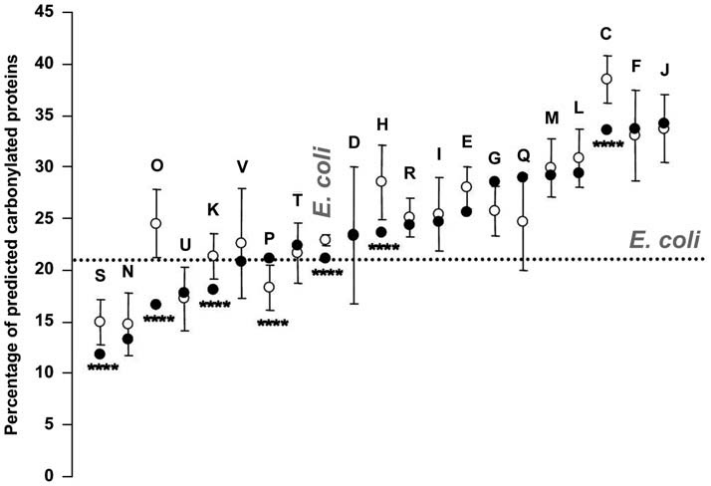
Percentage of predicted CP within functional COG classes. The predictive CSPD model was run on all protein classes, leading to the determination of the percentage of predicted CP for the standard proteome (Black circles) for each protein class. The average (open circle) and the standard deviations obtained for the 1000 shuffled proteomes (see Materials and Methods section) are indicated for each class. The asterisks highlight significant differences between shuffled and standard proteome in terms of percentages of predicted CP (p value <0.005). Designations of functional categories: C, energy production and conversion, D, cell cycle control and mitosis, E, amino acid metabolism and transport, F, nucleotide metabolism and transport, G, carbohydrate metabolism and transport, H, coenzyme metabolism, I, lipid metabolism, J, translation, K, transcription, L, replication and repair, M, cell wall/membrane/envelope biogenesis, N, cell motility, O, post-translational modification, protein turnover, chaperone functions, P, inorganic ion transport and metabolism, Q, secondary metabolite biosynthesis, transport and catabolism, R, general functional prediction only (typically, prediction of biochemical activity) S, unknown function, T, signal transduction, and U, intracellular trafficking and secretion, V, defense mechanisms. The dotted line represents the percentage of predicted CP for the whole standard *E. coli* proteome.

Next, we assigned for each protein a COG number (cluster of orthologous group) and functional class categories as described in Materials and Methods section. Data analysis showed differences in the percentages of predicted CP as a function of the class. Indeed, the lowest percentage (11.85%) is observed for protein of unknown function (class S) and the highest percentage (34.21%) for proteins involved in translation, ribosomal structure and biogenesis (class J) (note that a value of 21.18% is observed for the whole *E. coli* proteome). Also, proteins related to class C (energy, production and conversion), F (nucleotide transport and metabolism), and J (translation, ribosomal structure and biogenesis) were significantly enriched in predicted CP as compared with the mean value obtained for the *E. coli* proteome (Fig. 6). By contrast, among the COG classes with the lowest abundance of predicted CP, we identified proteins related to class N (cell motility) and O (postranslational modification, protein turnover, chaperones) (Fig. 6). These observations clearly indicate that the abundance of predicted CP is associated with the functional protein category. Moreover, the percentage of predicted CP for 12 out of 20 COG classes falls within the 95% confidence interval observed after shuffling (Fig. 6). More interestingly, in 6 COG classes (S, O, K, P, H, C) the percentage of predicted CP is significantly different (p<0.005) from the corresponding 95% confidence interval obtained after shuffling (Fig. 6). Enrichment in predicted CP was observed for proteins involved in inorganic ion transport and metabolism (class P), and depletion of predicted CP was observed for the 5 other COG classes (S, O, K, H, C). Finally, we also observed that the whole *E. coli* proteome had less predicted CP than the average value obtained with the 1000 shuffled proteomes.

## Discussion

It is commonly admitted that carbonylated proteins (CP) lose their functional activity both *in vitro* or *in vivo* [23]–[26]. Several groups have suggested that carbonylation acts as a tag for rapid proteolysis [23], [27], [28]. More generally, carbonylation is thought to be biologically significant, due to its irreversible nature. However, to date no rules have been found to predict sites or to understand the propensity of proteins to undergo carbonylation. Here, taking into account protein primary sequences and using both *in vitro* and *in vivo* approaches, we showed that some regions are more reactive to direct MCO attacks leading to carbonylation. Based on these rules hitherto uncovered, we developed a model allowing the prediction of sites more prone to carbonylation and hence of proteins more prone to carbonylation via direct MCO attack.

Based on the identification of specific carbonylated sites (CS) in oxidised BSA (*in vitro*) and in 23 *E. coli in vivo* CP, we have shown that carbonylation is highly selective, with only a few carbonylatable sites being effectively carbonylated. This observation is in agreement with the data already reported by several research groups [12], [14], [16]. We experimentally observed that, *in vitro* at the highest MCO level, around 80% of CS are solvent accessible. As the percentage of solvent accessible R, K, P, and T sites within BSA is close to 80%, our results suggest no over-representation of solvent accessible CS within carbonylatable sites. Interestingly however, at lower MCO levels, only solvent accessible CS were detected, indicating that these sites are more susceptible to carbonylation. Hence, at higher MCO levels, a partial local structural rearrangement due to carbonylation is likely to occur, thus leading to solvent exposure of previously buried carbonylatable sites.

Our *in vitro* and *in vivo* observations highlight the fact that the apparent reactivity of a carbonylatable site is greater if a neighbouring carbonylatable site (being as close as one amino acid away) is already carbonylated. Thus, once the first site is carbonylated, a spreading of oxidation will occur on neighbouring carbonylatable sites, resulting in several carbonylations via direct MCO attacks. Hydroxyl radicals, formed *via* the Fenton reaction, are the only ROS able to oxidise a carbonylatable site [29]. By consequence, potential iron binding sites close to carbonylatable sites promote the effective oxidation of these sites. Hence, the observed spreading of carbonylation suggests that several hydroxyl radicals are produced in close proximity to this region. In this context, iron atoms should be attracted by the environment around the carbonylatable site. Thus, it makes sense that RKPT-enriched regions in close proximity to a specific environment enriched in iron binding sites (D, E, Y, H, C) may be more reactive to direct MCO attacks. Notably, the generation of the first CS leads to the formation of a Lewis base, which is a new potential iron-binding site able to attract an iron atom. This new iron-binding site renders this region more responsive to iron and, by consequence, increases the chances of hydroxyl radical formation triggering the formation of the second CS. This schema leads to a positive feedback catastrophe propagation triggering carbonylation of all carbonylatable residues within an RKPT-enriched region.

Based on the observed spreading of carbonylation within RKPT-enriched regions and on the presence of a specific environment leading to a better reactivity for carbonylation, we suggest the first predictive model (predictive CSPD model) to allow the prediction of sites more prone to carbonylation, referred to as predicted HSC, and hence of proteins more prone to carbonylation via direct MCO attacks.

Around 10% of *E. coli* proteins detected by 2D electrophoresis analysis are experimentally found to be carbonylated [17], [19] when cells were cultivate without exogenous stress, but using the predictive CSPD model we showed that about 21% of the *E. coli* proteins are predicted to be carbonylated. Our predictive results raise the question whether this difference is due to (i) a limit in the detection sensitivity leading to an underestimation of carbonylation, or (ii) a rapid degradation of some CP [7] leading to an absence of detection by 2D electrophoresis analysis. In support of the latter hypothesis, we have recently shown that only CP in an aggregated and ‘less prone to degradation’ state could be detected, thus leading us to speculate that some CP escape identification, due to their high degradation rate [17].

Next, applying the predictive CSPD model to functional COG categories, we obtained predictions that were in good agreement with experimental results. For instance, ribosomal proteins (class J) and tricarboxylic acid (TCA) cycle enzymes (within class C), which are the most CP families in *E. coli* [7], [30], displayed the highest proportion of predicted CP (around 35%) (Fig. 6). Finally, we observed a large variation and heterogeneity in terms of percentage of predicted CP among the different protein families, suggesting that either protein families have evolved to over- or under-represent predicted HSC, or that the amino acid composition of these families predetermines the percentage of proteins containing predicted HSC. Shuffling analyses led us to conclude that among the 20 protein classes analysed, 14 families (N, U, V, T, D, R, I, E, G, Q, M, L, F) have a percentage of predicted CP (ranging from 15% to more than 30%) that is indeed predetermined by the amino acid composition. Conversely, for the six other families (S, O, K, P, H, C), the percentage of predicted CP (also ranging from 15% to more than 30%) is not predetermined solely by the amino acid composition. Altogether, these results suggest that proteins in *E. coli* in a sense have evolved to selectively maintain or lose predicted HSC according to their function. Interestingly, five functional classes (S, O, K, H, C) are depleted in proteins with predicted HSC. If we assume that the presence of a predicted HSC within a protein renders this protein more prone to carbonylation and by consequence more prone to degradation, we could speculate that these latter families have, in a sense, evolved to protect them against oxidation and degradation during oxidative stress. Interestingly, these families embrace chaperones and proteins involved in protein turnover and transcription, which are all involved in the defense against oxidative stress. Finally, the finding that proteins of the “natural” *E. coli* proteome have less predicted HSC than the shuffled proteome suggests that *E. coli* has evolved in a manner allowing its proteome to escape oxidative damage.

Notably, the efficiency of the predictive CSPD model in identifying CP was also confirmed upon analysis of the *B. subtilis* proteome. Strikingly, the predictive CSPD model failed to identify most CP experimentally detected in *A. thaliana* and *S. cerevisiae* proteomes. The inadequacy of the predictive CSPD model in identifying CP in these two organisms could be accounted for by the fact that some CP may result from secondary reactions with reactive carbonyl compounds on carbohydrates, lipids and advanced glycation/lipoxidation end products. The predictive CSPD model specifically only detects CP generated via direct MCO attacks. Thus, further efforts will be needed to uncover the rules and factors leading to the detection of CP generated via secondary reactions.

## Materials and Methods

### MCO of BSA

BSA (Sigma) was dissolved at 10 mg/ml in oxidation buffer (50 mM Hepes buffer, pH 7.4, containing 100 mM KCl and 10 mM MgCl_2_). Oxidation was accomplished by supplementing 750 µl of protein solution (7.5 mg) with a freshly prepared mixture of ascorbic acid/FeCl_3_ with final concentrations ranging from 25 10^−3^ mM/100 10^−3^ µM (named MCO level 0.001) to 25 mM/100 µM (named MCO level 1 - the most commonly used concentration for oxidation), respectively, and incubating overnight (15 h) at 37°C in a shaking bath. Oxidation was stopped by addition of 1 mM EDTA, and samples were dialyzed at 4°C against oxidation buffer supplemented with 1 mM EDTA. Protein concentration was determined with the bicinchoninic acid method (Pierce).

### SDS-PAGE and BSA western blots

SDS-PAGE of BSA was performed using a Mini-PROTEAN II electrophoresis cell (from Bio-Rad) with 10% (w/v) polyacrylamide resolving gel. Gels were stained with Coomassie Brilliant Blue R250, or processed immediately for immunoblotting onto a PVDF membrane using a semidry blotting system. A chemiluminescence kit (ECL plus, Amersham) was used to visualise and record the stained proteins.

### Carbonylation assays

Using an OxyBlot^TM^ protein oxidation detection kit (Chemicon International), carbonyl groups in the protein side chains were derivatised to 2,4-dinitrophenylhydrazone (DNP) by reaction with 2,4-dinitrophenylhydrazine (DNPH), as already described [23].

### Mass spectrometry identification of proteins

For protein identification, the silver-stained spots from 2D SDS-PAGE were excised from the gel and fully destained using ProteoSilver ^TM^ destainer Kit (Sigma). After several washes and drying by dehydration, samples were digested with trypsin (Promega, Madison, WI) as described previously [31]. BSA sample digestion was performed in liquid mixtures. Supernatant peptides and extraction washes were recovered and dried with a speed vacuum for the proteomic analysis performed by LC nano-ESI MS/MS. The nano High Pressure Liquid Chromatography instrument used in this study was a Finnigan Surveyor system (Thermo Electron, San Jose, CA, USA) equipped with a Spark micro AS autosampler and a Rheodyne ten-port switching valve and nano dynamic (NSI) probe assembly on an ion trap Finnigan LCQ-DECA ^XP^ spectrometer (Thermo Electron, San Jose, CA, USA).

The peptide mixture was dissolved in 5% formic acid in water and about 2/3 of the total volume were injected onto a Finnigan ProteomeX 2.0 workstation.

Peptides were separated on a reverse-phase PicoFrit^TM^ column (5 µm BioBasic C18, 300 Ä pore size, 75 µm×10 cm, tip 15 µm) (New Objective, Woburn, MA, USA). The peptides were ionised with a capillary temperature of 160°C and a 2.2 kV spray voltage. Three MS/MS spectra of the most intense peaks were obtained following one full scan mass spectrum (MS). The dynamic exclusion features were set at a repeat count of 2 within 0.5 min, with an exclusion duration of 3 min. For data analysis, protein identification was performed using the TurboSequest algorithm implemented within the Bioworks 3.1 software package (Thermo Electron Corporation) with non redundant NCBI *E. coli* database (release Sept. 2007, *E. coli* K12, 4320 sequences). The identified peptides were further evaluated using charge state versus cross-correlation number (*Xcorr*). The criteria for positive identification of peptides was Xcorr>1.5 for singly-charged ions, Xcorr>2.0 for doubly-charged ions and Xcorr>2.5 for triply-charged ions. Criteria for positive identification of oxidative modification are provided in supplementary Materials and Methods.

### Assessing or predicting the structural environment of CS

Whenever possible, assessment of the structural environment and of solvent exposure of predicted HSC and isolated CS was done by visual inspection of the relevant pdb files. Each protein sequence was therefore used as a query to browse the PDB using PDB-Blast [32]. For 21 out of 23 protein sequences, this led to the identification of a pdb file corresponding to either the structure of the query or to that of a homologous protein (see Table 3). For those cases where structural data were either completely (NP_415431.1, NP_414719.1) or partially lacking (NP_414555.1, NP_417799.1, NP_414596.1, NP_415477.1 and NP_418414.1), we carried out structural predictions aimed at assessing whether predicted HSC and isolated CS were located in ordered or disordered regions. Formally, disordered regions are defined as regions lacking a precise 3D structure and consisting of an ensemble of fluctuating, interconverting conformers.Predictions of (dis)order were carried out using the Metaserver of Disorder MeDor [33]. MeDor collects disorder and secondary structure predictions from servers available on the web and generates a graphical output. Specifically, it uses predictions from 10 disorder predictors, namely IUPred [34], Prelink [35], RONN [36], FoldUnfold [37], [38], DisEMBL [39], Foldindex [40], Globplot2 [39], Disprot VL3, Disprot VL3H [41], Disprot VSL2B [42], and performs secondary structure prediction using the pred2ary algorithm using the default parameters [43]. It also incorporates hydrophobic cluster analysis (HCA) [44] and generates a HCA plot. MeDor generates no automated consensus on (dis)order; thus, the assignment of order and disorder was done on a case-by-case basis, taking into account the length of the concerned region and the accuracy of the various predictors in identifying regions of short (<30 residues) or long (>70 residues) disorder (for reviews on the identification of disorder see [45], [46]. Coiled-coils, which correspond to regions that often fool some predictors into giving wrong predictions, were first identified by visual inspection of the HCA plot and then confirmed using the Multicoil program [47]. Hence, disorder predictions for such regions were considered poorly reliable. Small hydrophobic clusters occurring within mainly disordered regions, as observed in HCA plots, were taken as ordered, but they may correspond to regions able to fold only in the presence of a partner or ligand [45], [46].

### Computational analysis

#### Data sources and programs

The proteome sequences of *A. thaliana*, *E. coli* (strain K12), *B. subtilis* (*subtilis* str.168) and *S. cerevisiae* were downloaded from the NCBI ftp website (ftp://ftp.ncbi.nih.gov). The cai and shuffleseq programs (from the EMBOSS package, http://emboss.sourceforge.net) were used to determine the codon adaptation index (CAI) and to shuffle the protein sequence, respectively. In-house Perl scripts were used for computational analysis as well as for statistical calculations.

#### Principle of HSC and CP prediction

The predictive CSPD model (Fig. 3) is based on a set of rules, each composed of three parameters referred to as (X; Y; Z): X is the list of amino acids required during the detection process; Y is the detection cutoff (i.e. the minimun occurrence of the amino acid needed for the validation of the parameters); and Z is the detection window size. The predictive CSPD model runs as follows: (1) it first searches along the protein sequence for an RKPT-enriched region that consists of a local enrichment (3 out of 4 residues) of carbonylatable amino acids (R, K, P or T); and (2) the following step consists of searching for the presence of other amino acids (for the 6 environmental rules) in the proximity of or within the RKPT-enriched region. Detection of the enrichment of one or a set of amino acids in the environment and/or within an RKPT-enriched region is done with an initial window size of W, followed by window sliding (with +1 incrementations to the right) as described in Fig. 3. Note that in the sliding process, Wa and Wb are defined such that Wa + Wb  =  W. When the variable is validated (i.e containing the minimal number of amino acids required), E_L_ and E_R_ constitute the protein coordinates associated with the variable detection rules. Finally, when all of the rules are validated, this RKPT-enriched region is defined as a predicted HSC and, by consequence, a protein containing a predicted HSC becomes a predicted CP. HSC sequence position is therefore defined as HSC_L_ and HSC_R_, and the environment coordinates of the predicted HSC are defined as *min* (E_L_(*i*)) and *max* (E_R_ (*i*)) (where *i* is an environment factor). Note that several predicted HSC can be identified within the same protein sequence. If several predicted HSC are overlapped, they are fused, resulting in a predicted HSC of 5, 6, 7... or more amino acids. The CSPD model was developed for use as a web tool (in PHP programming language) (www.lcb-cnrs.mrs/CSPD/), allowing online predictions of CP and HSC.

#### Analysis of the HSC environment

To assess the possible existence of a common pattern associated with the HSC environment region, the following strategy was performed. (1) Amino acids of each environment sequence were re-coded as follows: K was replaced by R; V and I were replaced by A; and E, H, Y and C were replaced by D; except for P, G, T, A, D and R, other residues were replaced by X. (2) For each left position (position −n,...,−3,−2,−1) or for each right position (+1,+2,+3,....,+n) with respect to the HSC, the proportion of P, T, A, R, G, D and X was calculated for the overall HSC environment sequences.

#### Functional analysis of the predicted CP in E. coli

Functional analysis of the predicted CP in *E. coli* was performed as follows: (1) the COGnitor program [48], [49] was used to associate each protein with a COG (Cluster of Orthologous Genes) group and a defined functional category (or class). (2) The predictive CSPD model was then run on proteins in each class, leading to the estimation of the predicted CP percentage within the class (i. e. the number of predicted CP within a class over the number of proteins within the same class). (3) Subsequently, we applied a shuffling procedure to each COG class proteome as follows: (a) we shuffled the sequence of all the proteins within each COG class one thousand times, and therefore generated 1000 random proteomes for the same COG; (b) predicted CP were then identified using the predictive CSPD model, thus leading to the determination of a shuffled predicted CP percentage. (4) Finally, for each COG class, a statistical confidence interval was estimated. The same procedure was done using the standard *E. coli* proteome, leading to the determination of 1000 predicted CP percentage in *E. coli*.

#### Statistical evaluation of the performance of the detection model

The sensitivity and specificity of predicitive CSPD model were calculated as TP/(TP+FN) and TN/(FP+TN), respectively where TP represents True Positives (*i.e.* the number of detected proteins from the set of CP); FP, False Positives (*i.e*. the number of detected proteins that are not part of the set of CP); FN, False Negative (i.e. the number of non-detected proteins from the set of CP); and TN, True Negative (*i.e.* the number of non-detected proteins that are not part of the set of CP). Precision (measure of the exactness of the detection) or predictive positive value of protein detection was computed as TP/(TP+FP).

## Supporting Information

Figure S1 MS spectra from MALDI TOF analysis of BSA at different MCO levels. Arrows point out the appearance of BSA fragmentation and the BSA dimer disappearance as the MCO level increases.
(0.11 MB DOC)

Figure S2 MS/MS spectra from peptide R459 to L483. MS/MS spectrum of the five peptides, containing 0 to 7 oxidations, with overlapping RKPT-enriched regions (T462 to T472). (A) MS/MS spectrum for peptide P464 to R482 and for peptide P464 until R482 (B) confirming that P464 and P470 were oxidised and DNP-labelled with a mass difference of +196 Da. (C) MS/MS spectrum for peptide P464 to R482 confirming that P464 M469 and P470 were oxidised with a mass difference of +16 Da. (D) MS/MS spectrum for peptide P464 to R482 confirming that P464 R468 M469 and P470 were oxidised with a mass difference of +16, -43, +16, +16 Da, respectively. (E) MS/MS spectrum for peptide C460 to R482 confirming that T462, K463, P464, R468, M469, P470 and R482 were oxidised with a mass difference of +178, +179 +196, +137, +16, +196 and +137 Da, respectively.
(0.34 MB DOC)

Figure S3 Carbonyl content 2D electrophoresis analysis of unsoluble cell fractions from exponentially grown E. coli. (A) Specific pattern of carbonylation in unsoluble cell fractions from exponentially grown E. coli, as determined by two-dimensional western blot immunoassays, carried out as previously described [1]. (B) PVDF membrane after 2D western blot stained with Coomassie blue. (C) Silver staining after 2D gel electrophoresis.
(0.62 MB DOC)

Figure S4 Site-specific carbonylation in E. coli proteins. Amino acid sequences of E. coli CP were obtained from the NCBI database. CS, based on mass spectrometry analysis, are highlighted in yellow. The regions of the sequence identified by mass spectrometry are shown in red. RPKT-enriched regions (3 carbonylatable sites within a sequence of 4 amino acids) are framed. Predicted HSC are shown in bold. CS are shaded in yellow.
(0.09 MB DOC)

Figure S5 Analysis of the amino acid environment around predicted HSC. Occurence of amino acid subgroups in regions flanking a predicted HSC. Amino acid subgroups are (D, E, H, Y, C) (violet), (A, V, G, I) (cyan) (P, T) (pink), and (G) (yellow). The dotted line shows the average occurence of the corresponding amino acid subgroups in the E. coli proteome.
(0.09 MB DOC)

Table S1 Parameters for the identification of RKPT-enriched regions The primary sequence of BSA contains an average of one carbonylatable site within a sequence of 4 amino acids (23.5% of carbonylatable sites); thus, we tested how to define an RKPT-enriched region by analysing the specificity, the sensitivity and the positive predictive value of four enrichments, from 1 to 4 carbonylatable sites, within a sequence of 4 amino acids.
(0.03 MB DOC)

Table S2 MS data for MALDI-TOF global mass analysis of BSA (*) M/Z of BSA monomer were obtained with standard deviations of 1000 ppm (▒) As the ionisation energy was the same for each sample, the half height width of the BSA monomer accounted for the BSA molecule heterogeneity. However, due to the quantitative decrease of BSA monomer (for instance at MCO level 1), the signal to noise ratio decreased leading to an over-estimation of the height width of the BSA monomer.
(0.03 MB DOC)

Table S3 Data analysis of CS found in the proteins analysed by nano-LC ESI MS/MS. List of all carbonylated peptides containing at least one CS as judged by mass spectrometry analysis. CS and oxidised methionines are shaded in gray. Whenever possible, assessment of the structural environment and of the solvent exposure of isolated CS was done by visual inspection of the relevant pdb or homologue files. CS were thus mapped within (1) apha helices, (2) loops, and (3) beta strands. Asterisks (*) show CS not conserved in the corresponding PDB homologue. For those cases where structural data were either completely ▒ (NP_415431.1, NP_414719.1) or partially lacking (NP_414555.1, NP_417799.1, NP_414596.1, NP_415477.1 and NP_418414.1), we carried out structural predictions to locate residues in ordered or disordered (see experimental procedures).
(0.08 MB DOC)

Table S4 Set of E. coli proteins specifically carbonylated or uncarbonylated. The table shows three sets of proteins used to test the efficiency of the CSPD model. CP from exponentially grown cells were provided from this study. CP from the stationary phase were obtained from several studies already carried out on E. coli [1], [2]. Non-CP were provided from this study. Proteins containing at least one predicted HSC are indicated by an asterisk. [1] Dukan S, Nystrom T (1998) Bacterial senescence: stasis results in increased and differential oxidation of cytoplasmic proteins leading to developmental induction of the heat shock regulon. Genes Dev 12: 3431–3441. [2] Dukan S, Nystrom T (1999) Oxidative stress defense and deterioration of growth-arrested Escherichia coli cells. J Biol Chem 274: 26027-26032.
(0.07 MB DOC)
