# Supplementary figures and images for: Rules Governing Selective Protein Carbonylation
Source: PLoS One. 2009 Oct 5;4(10):e7269. doi: 10.1371/journal.pone.0007269 (PMC2751825; doi:10.1371/journal.pone.0007269)

**Figure S1**

**
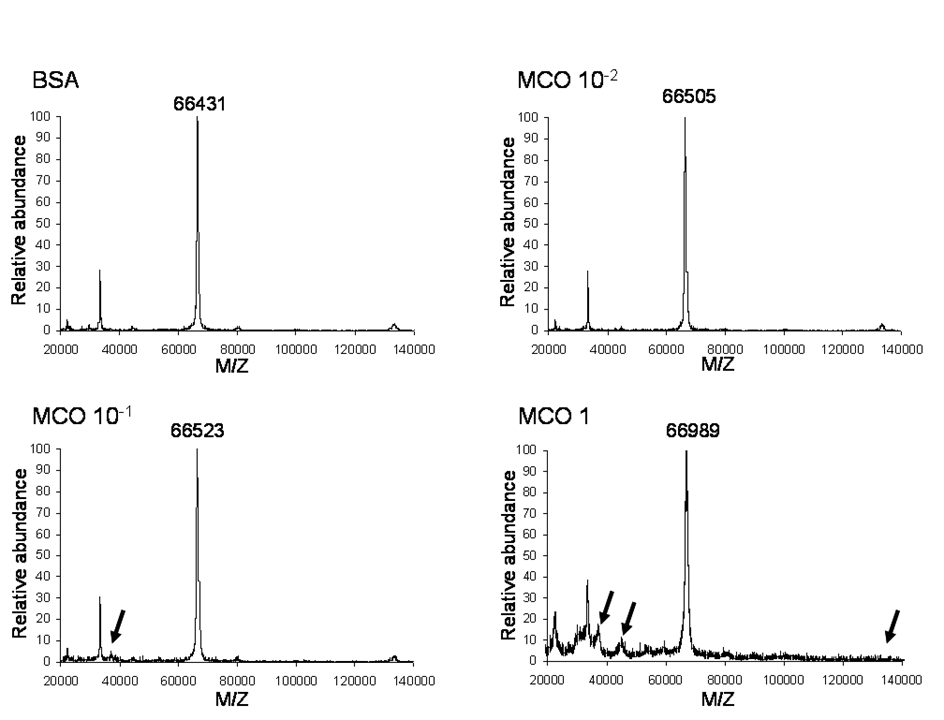
**

Supplement: Figure S1 — MS spectra from MALDI TOF analysis of BSA at different MCO levels. Arrows point out the appearance of BSA fragmentation and the BSA dimer disappearance as the MCO level increases. (0.11 MB DOC) [file pone.0007269.s001.doc]

**Figure S2**

**
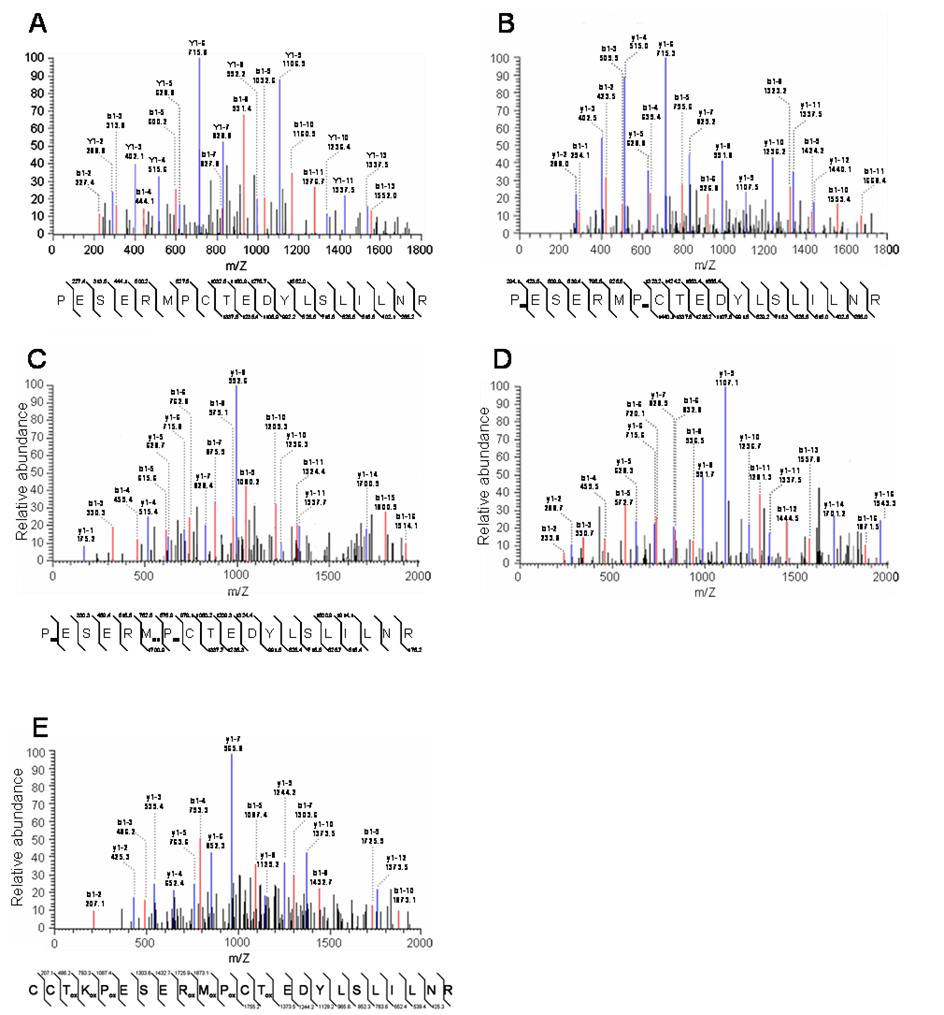
**

Supplement: Figure S2 — MS/MS spectra from peptide R459 to L483. MS/MS spectrum of the five peptides, containing 0 to 7 oxidations, with overlapping RKPT-enriched regions (T462 to T472). (A) MS/MS spectrum for peptide P464 to R482 and for peptide P464 until R482 (B) confirming that P464 and P470 were oxidised and DNP-labelled with a mass difference of +196 Da. (C) MS/MS spectrum for peptide P464 to R482 confirming that P464 M469 and P470 were oxidised with a mass difference of +16 Da. (D) MS/MS spectrum for peptide P464 to R482 confirming that P464 R468 M469 and P470 were oxidised with a mass difference of +16, -43, +16, +16 Da, respectively. (E) MS/MS spectrum for peptide C460 to R482 confirming that T462, K463, P464, R468, M469, P470 and R482 were oxidised with a mass difference of +178, +179 +196, +137, +16, +196 and +137 Da, respectively. (0.34 MB DOC) [file pone.0007269.s002.doc]

**
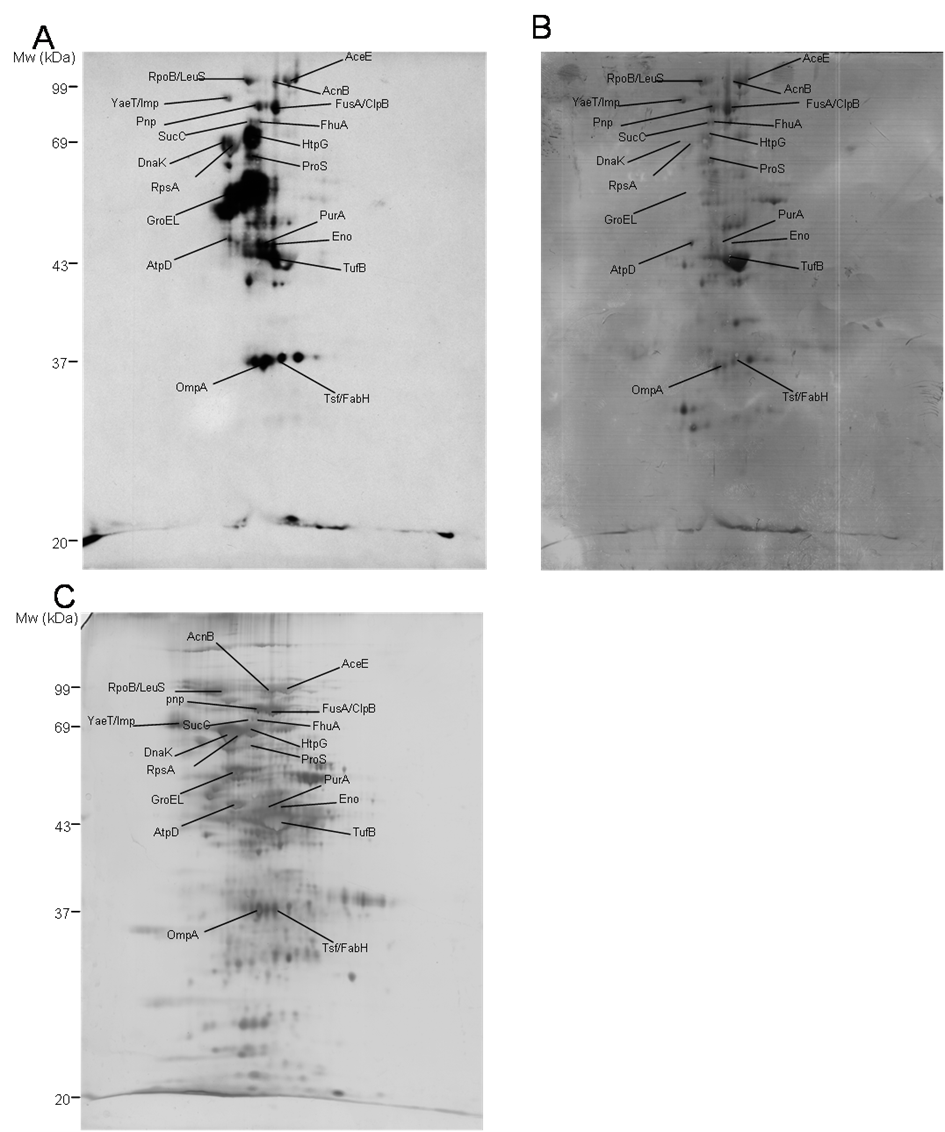
**

Supplement: Figure S3 — Carbonyl content 2D electrophoresis analysis of unsoluble cell fractions from exponentially grown E. coli. (A) Specific pattern of carbonylation in unsoluble cell fractions from exponentially grown E. coli, as determined by two-dimensional western blot immunoassays, carried out as previously described [1]. (B) PVDF membrane after 2D western blot stained with Coomassie blue. (C) Silver staining after 2D gel electrophoresis. (0.62 MB DOC) [file pone.0007269.s003.doc]

**Figure S5**

**
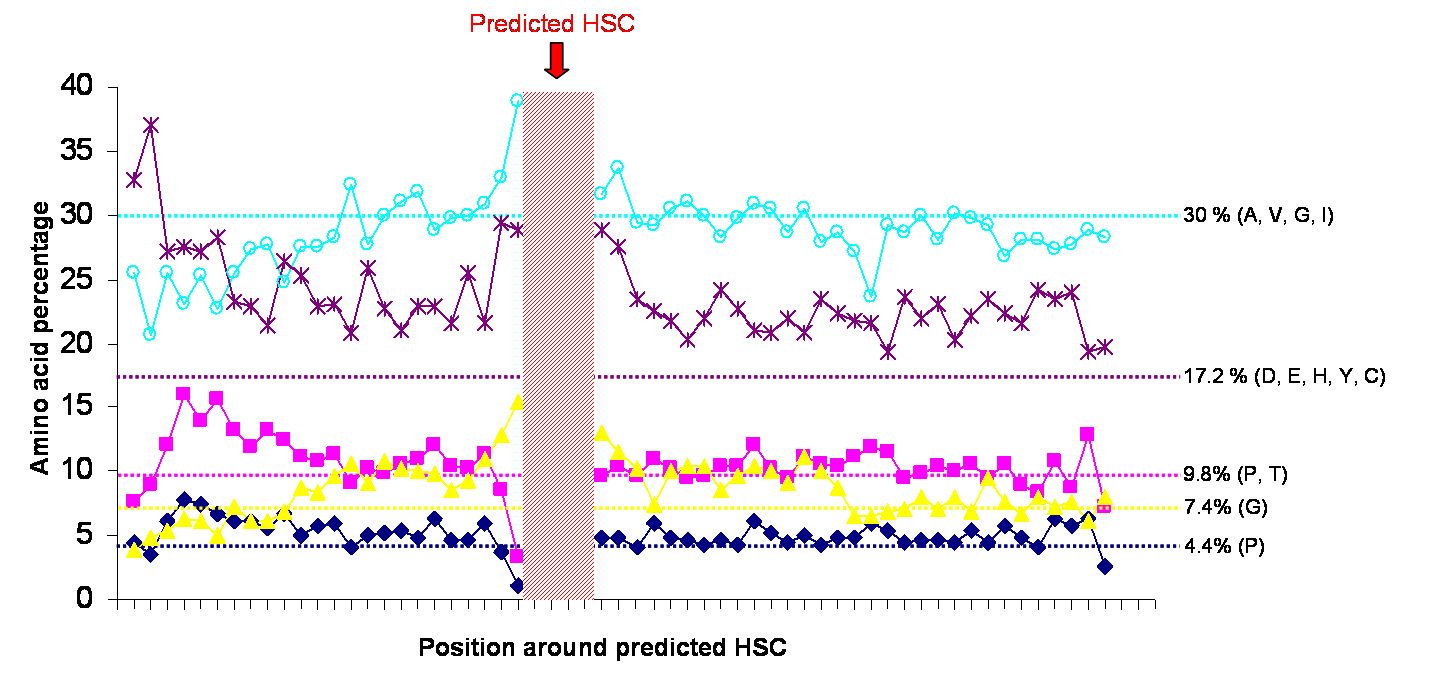
**

Supplement: Figure S5 — Analysis of the amino acid environment around predicted HSC. Occurence of amino acid subgroups in regions flanking a predicted HSC. Amino acid subgroups are (D, E, H, Y, C) (violet), (A, V, G, I) (cyan) (P, T) (pink), and (G) (yellow). The dotted line shows the average occurence of the corresponding amino acid subgroups in the E. coli proteome. (0.09 MB DOC) [file pone.0007269.s005.doc]
