# Supplementary material for: Rules Governing Selective Protein Carbonylation
Source: PLoS One. 2009 Oct 5;4(10):e7269. doi: 10.1371/journal.pone.0007269 (PMC2751825; doi:10.1371/journal.pone.0007269)
Supplement: Figure S4 — Site-specific carbonylation in E. coli proteins. Amino acid sequences of E. coli CP were obtained from the NCBI database. CS, based on mass spectrometry analysis, are highlighted in yellow. The regions of the sequence identified by mass spectrometry are shown in red. RPKT-enriched regions (3 carbonylatable sites within a sequence of 4 amino acids) are framed. Predicted HSC are shown in bold. CS are shaded in yellow. (0.09 MB DOC) [file pone.0007269.s004.doc]

Figure S4

**AceE**

**>gi|2506964|sp|P06958|ODP1_ECOL: 887 Pyruvate dehydrogenase E1 component**

MSERFPNDVDPIETRDWLQAIESVIREEGVERAQYLIDQLLAEARKGGVNVAAGTGISNYINTIPVEEQPEYPGNLELERRIRSAIRWNAIMTVLRASKKDLELGGHMASFQSSATIYDVCFNHFFRARNEQDGGDLVYFQGHISPGVYARAFLEGRLTQEQLDNFRQEVHGNGLSSYPHPKLMPEFWQFPTVSMGLGPIGAIYQAKFLKYLEHRGLKDTSKQTVYAFLGDGEMDEPESKGAITIATREKLDNLVFVINCNLQRLDGPVTGNGKIINELEGIFEGAGWNVIKVMWGSRWDELLRKDTSGKLIQLMNETVDGDYQTFKSKDGAYVREHFFGKYPETAALVADWTDEQIWALNRGGHD**PKKI**YAAFKKAQETKGKATVILAHTIKGYGMGDAAEGKNIAHQVKKMNMDGVRHIRDRFNVPVSDADIEKLPYITFPEGSEEHTYLHAQRQKLHGYLPSRQPNFTEKLELPSLQDFGALLEEQSKEISTTIAFVRALNVMLKNKSIKDRLVPIIADEARTFGMEGLFRQIGIYSPNGQQYTPQDREQVAYYKEDEKGQILQEGINELGAGCSWLAAATSYSTNNLPMIPFYIYYSMFGFQRIGDLCWAAGDQQARGFLIGGTSGRTTLNGEGLQHEDGHSHIQSLTIPNCISYDPAYAYEVAVIMHDGLERMYGEKQENVYYYITTLNENYHMPAMPEGAEEGIRKGIYKLETIEGSKGKVQLLGSGSILRHVREAAEILAKDYGVGSDVYSVTSFTELARDGQDCERWNMLHPLETPRVPYIAQVMNDAPAVASTDYMKLFAEQVRTYVPADDYRVLGTDGFGRSDSRENLRHHFEVDASYVVVAALGELAKRGEIDKKVVADAIAKFNIDADKVNPRLA

**AcnB**

**>gi|2506132|sP|P36683|ACON2_ECOL: 865 Aconitate hydratase 2 (Citrate hydro-lyase 2) (Aconitase 2)**

MLEEYRKHVAERAAEGIAPKPLDANQMAALVELLKNPPAGEEEFLLDLLTNRVPPGVDEAAYVKAGFLAAIAKGEAKSPLLTPEKAIELLGTMQGGYNIHPLIDALDDAKLAPIAAKALSHTLLMFDNFYDVEEKAKAGNEYAKQVMQSWADAEWFLNRPALAEKLTVTVFKVTGETNTDDLSPAPDAWSRPDIPLHALAMLKNAREGIEPDQPGVVGPIKQIEALQQKGFPLAYVGDVVGTGSSRKSATNSVLWFMGDDIPHVPNKRGGGLCLGGKIAPIFFNTMEDAGALPIEVDVSNLNMGDVIDVYPYKGEVRNHETGELLATFELKTDVLIDEVRAGGRIPLIIGRGLTTKAREALGLPHSDVFRQAKDVAESDRGFSLAQKMVGRACGVKGIRPGAYCE**PKMT**SVGSQD**TTGPMTR**DELKDLACLGFSADLVMQSFCHTAAYPKPVDVNTHHTLPDFIMNRGGVSLRPGDGVIHSWLNRMLLPDTVGTGGDSHTRFPIGISFPAGSGLVAFAAATGVMPLDMPESVLVRFKGKMQPGITLRDLVHAIPLYAIKQGLLTVEKKGKKNIFSGRILEIEGLPDLKVEQAFELTDASAERSAAGCTIKLNKEPIIEYLNSNIVLLKWMIAEGYGDRRTLERRIQGMEKWLANPELLEADADAEYAAVIDIDLADIKEPILCAPNDPDDARPLSAVQGEKIDEVFIGSCMTNIGHFRAAGKLLDAHKGQLPTRLWVAPPTRMDAAQLTEEGYYSVFGKSGARIEIPGCSLCMGNQARVADGATVVSTSTRNFPNRLGTGANVFLASAELAAVAALIGKLPTPEEYQTYVAQVDKTAVDTYRYLNFNQLSQYTEKADGVIFQTAV

**AtpD**

**>gi|114546|sp|P00824|ATPB_ECOLI ATP synthase beta chain**

MATGKIVQVIGAVVDVEFPQDAVPRVYDALEVQNGNERLVLEVQQQLGGGIVRTIAMGSSDGLRRGLDVKDLEHPIEVPVGKATLGRIMNVLGEPVDMKGEIGEEERWAIHRAAPSYEELSNSQELLETGIKVIDLMCPFAKGGKVGLFGGAGVGKTVNMMELIRNIAIEHSGYSVFAGVGERTREGNDFYHEMTDSNVIDKVSLVYGQMNEPPGNRLRVALTGLTMAEKFRDEGRDVLLFVDNIYRYTLAGTEVSALLGRMPSAVGYQPTLAEEMGVLQERITSTKTGSITSVQAVYVPADDLTDPSPATTFAHLDATVVLSRQIASLGIYPAVDPLDSTSRQLDPLVVGQEHYDTARGVQSILQRYQELKDIIAILGMDELSEEDKLVVARARKIQRFLSQPFFVAEVFTGSPGKYVSLKDTIRGFKGIMEGEYDHLPEQAFYMVGSIEEAVEKAKKL

**ClpB**

**>gi|54036848|sp|P63284|CLPB_ECOLI Chaperone clpB (Heat-shock protein F84.1)**

MRLDRLTNKFQLALADAQSLALGHDNQFIEPLHLMSALLNQEGGSVSPLLTSAGINAGQLRTDINQALNRLPQVEGTGGDVQPSQDLVRVLNLCDKLAQKRGDNFISSELFVLAALESRGTLADILKAAGATTANITQAIEQMRGGESVNDQGAEDQRQALKKYTIDLTERAEQGKLDPVIGRDEEIRRTIQVLQRRTKNNPVLIGEPGVGKTAIVEGLAQRIINGEVPEGLKGRRVLALDMGALVAGAKYRGEFEERLKGVLNDLAKQEGNVILFIDELHTMVGAGKADGAMDAGNMLKPALARGELHCVGATTLDEYRQYIEKDAALERRFQKVFVAEPSVEDTIAILRGLKERYELHHHVQITDPAIVAAATLSHRYIADRQLPDKAIDLIDEAASSIRMQIDSKPEELDRLDRRIIQLKLEQQALMKESDEASKKRLDMLNEELSDKERQYSELEEEWKAEKASLSGTQTIKAELEQAKIAIEQARRVGDLARMSELQYGKIPELEKQLEAATQLEGKTMRLLRNKVTDAEIAEVLARWTGIPVSRMMESEREKLLRMEQELHHRVIGQNEAVDAVSNAIRRSRAGLADPNRPIGSFLFLGPTGVGKTELCKALANFMFDSDEAMVRIDMSEFMEKHSVSRLVGAPPGYVGYEEGGYLTEA**VRRRPY**SVILLDEVEKAHPDVFNILLQVLDDGRLTDGQGRTVDFRNTVVIMTSNLGSDLIQERFGELDYAHMKELVLGVVSHNFRPEFINRIDEVVVFHPLGEQHIASIAQIQLKRLYKRLEERGYEIHISDEALKLLSENGYDPVYGARPLKRAIQQQIENPLAQQILSGELVPGKVIRLEVNEDRIVAVQ

**dnaK**

**>gi|12512692|gb|AAG54314.1|AE005178_: 630 chaperone Hsp70; DNA biosynthesis; autoregulated heat shock proteins [Escherichia coli O157:H7 EDL933]**

MGKIIGIDLGTTNSCVAIMD**GTTPRV**LENAEGD**RTTPS**IIAYTQDGETLVGQPAKRQAVTNPQNTLFAIKRLIGRRFQDEEVQRDVSIMPFKIIAADNGDAWVEVKGQKMAPPQISAEVLKKMKKTAEDYLGEPVTEAVITVPAYFNDAQRQATKDAGRIAGLEVKRIINEPTAAALAYGLDKGTGNRTIAVYDLGGGTFDISIIEIDEVDGEKTFEVLATNGDTHLGGEDFDSRLINYLVEEFKKDQGIDLRNDPLAMQRLKEAAEKAKIELSSAQQTDVNLPYITADATGPKHMNIKVTRAKLESLVEDLVNRSIEPLKVALQDAGLSVSDIDDVILVGGQTRMPMVQKKVAEFFGKEPRKDVNPDEAVAIGAAVQGGVLTGDVKDVLLLDVTPLSLGIETMGGVMTTLIAKNTTIPTKHSQVFSTAEDNQSAVTIHVLQGERKRAADNKSLGQFNLDGINPAPRGMPQIEVTFDIDADGILHVSAKDKNSGKEQKITIKASSGLNEDEIQKMVRDAEANAEADRKFEELVQTRNQGDHLLHSTRKQVEEAGDKLPADDKTAIESALTALETALKGEDKAAIEAKMQELAQVSQKLMEIAQQQHAQQQTAGADASANNAKDDDVVDAEFEEVKDKK

**Eno**

**>gi|68566315|sp|P0A6P9|ENO_ECOLI Enolase (2-phosphoglycerate dehydratase) (2-phospho-D-glycerate hydro-lyase)**

MSKIVKIIGREIIDSRGNPTVEAEVHLEGGFVGMAAAPSGASTGSREALELRDGDKSRFLGKGVTKAVAAVNGPIAQALIGKDAKDQAGIDKIMIDLDGTENKSKFGANAILAVSLANAKAAAAAKGMPLYEHIAELNG**TPGK**YSMPVPMMNIINGGEHADNNVDIQEFMIQPVGAKTVKEAIRMGSEVFHHLAKVLKAKGMNTAVGDEGGYAPNLGSNAEALAVIAEAVKAAGYELGKDITLAMDCAASEFYKDGKYVLAGEGNKAFTSEEFTHFLEELTKQYPIVSIEDGLDESDWDGFAYQTKVLGDKIQLVGDDLFVTNTKILKEGIEKGIANSILIKFNQIGSLTETLAAIKMAKDAGYTAVISHRSGETEDATIADLAVGTAAGQIKTGSMSRSDRVAKYNQLIRIEEALGEKAPYNGRKEIKGQA

**FabH**

**>gi|68566320|sp|P0A6R0|FABH_ECOLI 3-oxoacyl-[acyl-carrier-protein] synthase 3 (3-oxoacyl-[acyl-carrier-protein] synthase III) (Beta-ketoacyl-ACP synthase III) (KAS III) (EcFabH)**

MYTKIIGTGSYLPEQVRTNADLEKMVDTSDEWIVTRTGIRERHIAAPNETVSTMGFEAATRAIEMAGIEKDQIGLIVVATTSATHAFPSAACQIQSMLGIKGCPAFDVAAACAGFTYALSVADQYVKSGAVKYALVVGSDVLARTCDPTDRGTIIIFGDGAGAAVLAASEEPGIISTHLHADGSYGELLTLPNADRVNPENSIHLTMAGNEVFKVAVTELAHIVDETLAANNLDRSQLDWLVPHQANLRIISATAKKLGMSMDNVVVTLDRHGNTSAASVPCALDEAVRDGRIKPGQLVLLEAFGGGFTWGSALVRF

**FhuA**

**>gi|2507464|sp|P06971|FHUA_ECOLI Ferrichrome-iron receptor precursor (Ferric hydroxamate uptake) (Ferric hydroxamate receptor)**

MARSKTAQPKHSLRKIAVVVATAVSGMSVYAQAAVEPKEDTITVTAAPAPQESAWGPAATIAARQSATGTKTDTPIQKVPQSISVVTAEEMALHQPKSVKEALSYTPGVSVGTRGASNTYDHLIIRGFAAEGQSQNNYLNGLKLQGNFYNDAVIDPYMLERAEIMRGPVSVLYGKSSPGGLLNMVSKRPTTEPLKEVQFKAGTDSLFQTGFDFSDSLDDDGVYSYRLTGLARSANAQQKGSEEQRYAIAPAFTWRPDDKTNFTFLSYFQNEPETGYYGWLPKEGTVEPLPNG**KRLPT**DFNEGAKNNTYSRNEKMVGYSFDHEFNDTFTVRQNLRFAENKTSQNSVYGYGVCSDPANAYSKQCAALAPADKGHYLARKYVVDDEKLQNFSVDTQLQSKFATGDIDHTLLTGVDFMRMRNDINAWFGYDDSVPLLNLYNPVNTDFDFNAKDPANSGPYRILNKQKQTGVYVQDQAQWDKVLVTLGGRYDWADQESLNRVAGTTDKRDDKQFTWRGGVNYLFDNGVTPYFSYSESFEPSSQVGKDGNIFAPSKGKQYEVGVKYVPEDRPIVVTGAVYNLTKTNNLMADPEGSFFSVEGGEIRARGVEIEAKAALSASVNVVGSYTYTDAEYTTDTTYKGNTPAQVPKHMASLWADYTFFDGPLSGLTLGTGGRYTGSSYGDPANSFKVGSYTVVDALVRYDLARVGMAGSNVALHVNNLFDREYVASCFNTYGCFWGAERQVVATATFRF

**FusA**

**>gi|62288080|sp|P0A6M8|EFG_ECOLI Elongation factor G (EF-G)**

MARTTPIARYRNIGISAHIDAGKTTTTERILFYTGVNHKIGEVHDGAATMDWMEQEQERGITITSAATTAFWSGMAKQYEPHRINIIDTPGHVDFTIEVERSMRVLDGAVMVYCAVGGVQPQSETVWRQANKYKVPRIAFVNKMDRMGANFLKVVNQIKTRLGANPVPLQLAIGAEEHFTGVVDLVKMKAINWNDADQGVTFEYEDIPADMVELANEWHQNLIESAAEASEELMEKYLGGEELTEAEIKGALRQRVLNNEIILVTCGSAFKNKGVQAMLDAVIDYLPSPVDVPAINGILDDG**KDTP**AERHASDDEPFSALAFKIATDPFVGNLTFFRVYSGVVNSGDTVLNSVKAARERFGRIVQMHANKREEIKEVRAGDIAAAIGLKDVTTGDTLCDPDAPIILERMEFPEPVISIAV**EPKTK**ADQEKMGLALGRLAKEDPSFRVWTDEESNQTIIAGMGELHLDIIVDRMKREFNVEANVGKPQVAYRETIRQKVTDVEGKHAKQSGGRGQYGHVVIDMYPLEPGSNPKGYEFINDIKGGVIPGEYIPAVDKGIQEQLKAGPLAGYPVVDMGIRLHFGSYHDVDSSELAFKLAASIAFKEGFKKAKPVLLEPIMKVEVETPEENTGDVIGDLSRRRGMLKGQESEVTGVKIHAEVPLSEMFGYATQLRSLTKGRASYTMEFLKYDEAPSNVAQAVIEARGK

**GroE**

**sp|P0A6F5|CH60_ECOL: 547 60 kDa chapeRonin (PRotein Cpn60) (gRoEL pRotein) - EscheRichia coli.**

AAKDVKFGNDARVKMLRGVNVLADAVKVTLGPKGRNVVLDKSFGAPTITKDGVSVAREIELEDKFENMGAQMVKEVASKANDAAGDGTTTATVLAQAIITEGLKAVAAGMNPMDLKRGIDKAVTAAVEELKALSVPCSDSKAIAQVGTISANSDETVGKLIAEAMDKVGKEGVITVEDGTGLQDELDVVEGMQFDRGYLSPYFIN**KPET**GAVELESPFILLADKKISNIREMLPVLEAVAKAGKPLLIIAEDVEGEALATLVVNTMRGIVKVAAVKAPGFGDRRKAMLQDIATLTGGTVISEEIGMELEKATLEDLGQAKRVVINKDTTTIIDGVGEEAAIQGRVAQIRQQIEEATSDYDREKLQERVAKLAGGVAVIKVGAATEVEMKEKKARVEDALHATRAAVEEGVVAGGGVALIRVASKLADLRGQNEDQNVGIKVALRAMEAPLRQIVLNCGEEPSVVANTVKGGDGNYGYNAATEEYGNMIDMGIL**DPTKV**TRSALQYAASVAGLMITTECMVTDLPKNDAADLGAAGGMGGMGGMGGMM

**HtpG**

**>gi|16128457|ref|NP_415006.1: 624 heat shock protein 90 [Escherichia coli K12]**

MKGQETRGFQSEVKQLLHLMIHSLYSNKEIFLRELISNASDAADKLRFRALSNPDLYEGDGELRVRVSFDKDKRTLTISDNGVGMTRDEVIDHLGTIAKSGTKSFLESLGSDQAKDSQLIGQFGVGFYSAFIVADKVTVRTRAAGEKPENGVFWESAGEGEYTVADITKEDRGTEITLHLREGEDEFLDDWRVRSIISKYSDHIALPVEIEKREEKDGETVISWEKINKAQALWTRNKSEITDEEYKEFYKHIAHDFNDPLTWSHNRVEGKQEYTSLLYIPSQAPWDMWNRDHKHGLKLYVQRVFIMDDAEQFMPNYLRFVRGLIDSSDLPLNVSREILQDSTVTRNLRNALTKRVLQMLEKLAKDDAEKYQTFWQQFGLVLKEGPAEDFANQEAIAKLLRFASTHTDSSAQTVSLEDYVSRMKEGQEKIYYITADSYAAAKSSPHLELLRKKGIEVLLLSDRIDEWMMNYLTEFDGKPFQSVSKVDESLEKLADEVDESAKEAEKALTPFIDRVKALLGERVKDVRLTHRLTDTPAIVSTDADEMSTQMAKLFAAAGQKVPEVKYIFELNPDHVLVKRAADTEDEAKFSEWVELLLDQALLAERGTLEDPNLFIRRMNQLLVS

**Imp**

**>gi|2507089|sp|P31554|OSTA_ECOLI Organic solvent tolerance protein precursor**

MKKRIPTLLATMIATALYSQQGLAADLASQCMLGVPSYDRPLVQGDTNDLPVTINADHAKGDYPDDAVFTGSVDIMQGNSRLQADEVQLHQKEAPGQPE**PVRT**VDALGNVHYDDNQVILKGPKGWANLNTKDTNVWEGDYQMVGRQGRGKADLMKQRGENRYTILDNGSFTSCLPGSDTWSVVGSEIIHDREEQVAEIWNARFKVGPVPIFYSPYLQLPVGDKRRSGFLIPNAKYTTTNYFEFYLPYYWNIAPNMDATITPHYMHRRGNIMWENEFRYLSQAGAGLMELDYLPSDKVYEDEHPNDDSSRRWLFYWNHSGVMDQVWRFNVDYTKVSDPSYFNDFDNKYGSSTDGYATQKFSVGYAVQNFNATVSTKQFQVFSEQNTSSYSAEPQLDVNYYQNDVGPFDTRIYGQAVHFVNTRDDMPEATRVHLEPTINLPLSNNWGSINTEAKLLATHYQQTNLDWYNSRNTTKLDESVNRVMPQFKVDGKMVFERDMEMLAPGYTQTLEPRAQYLYVPYRDQSDIYNYDSSLLQSDYSGLFRDRTYGGLDRIASANQVTTGVTSRIYDDAAVERFNISVGQIYYFTESRTGDDNITWENDDKTGSLVWAGDTYWRISERWGLRGGIQYDTRLDNVATSNSSIEYRRDEDRLVQLNYRYASPEYIQATLPKYYSTAEQYKNGISQVGAVASWPIADRWSIVGAYYYDTNANKQADSMLGVQYSSCCYAIRVGYERKLNGWDNDKQHAVYDNAIGFNIELRGLSSNYGLGTQEMLRSNILPYQNTL

**LeuS**

**>gi|2507435|sp|P07813|SYL_ECOLI Leucyl-tRNA synthetase (Leucine--tRNA ligase) (LeuRS)**

MQEQYRPEEIESKVQLHWDEKRTFEVTEDESKEKYYCLSMLPYPSGRLHMGHVRNYTIGDVIARYQRMLGKNVLQPIGWDAFGLPAEGAAVKNNTAPAPWTYDNIAYMKNQLKMLGFGYDWSRELA**TCTP**EYYRWEQKFFTELYKKGLVYKKTSAVNWCPNDQTVLANEQVIDGCCWRCDTKVERKEIPQWFIKITAYADELLNDLDKLDHWPDTVKTMQRNWIGRSEGVEITFNVNDYDNTLTVY**TTRP**DTFMGCTYLAVAAGHPLAQKAAENNPELAAFIDECRNTKVAEAEMATMEKKGVDTGFKAVHPLTGEEIPVWAANFVLMEYGTGAVMAVPGHDQRDYEFASKYGLNIKPVILAADGSEPDLSQQALTEKGVLFNSGEFNGLDHEAAFNAIADKLTAMGVGERKVNYRLRDWGVSRQRYWGAPIPMVTLEDGTV**MPTP**DDQLPVILPEDVVMDGITSPIKADPEWAKTTVNGMPALRETDTFDTFMESSWYYARYTCPQYKEGMLDSEAANYWLPVDIYIGGIEHAIMHLLYFRFFHKLMRDAGMVNSDEPAKQLLCQGMVLADAFYYVGENGERNWVSPVDAIVERDEKGRIVKAKDAAGHELVYTGMSKMSKSKNNGIDPQVMVERYGADTVRLFMMFASPADMTLEWQESGVEGANRFLKRVWKLVYEHTAKGDVAALNVDALTENQKALRRDVHKTIAKVTDDIGRRQTFNTAIAAIMELMNKLAKAPTDGEQDRALMQEALLAVVRMLN**PFTP**HICFTLWQELKGEGDIDNAPWPVADEKAMVEDSTLVVVQVNGKVRAKITVPVDATEEQVRERAGQEHLVAKYLDGVTVRKVIYVPGKLLNLVVG

**OmpA**

**>sp|P0A910|OMPA_ECOL: 346 Outer membrane protein A precursor (Outer membrane protein II*) - Escherichia coli.**

MKKTAIAIAVALAGFATVAQAAPKDNTWYTGAKLGWSQYHDTGFINNNGPTHENQLGAGAFGGYQVNPYVGFEMGYDWLGRMPYKGSVENGAYKAQGVQLTAKLGYPITDDLDIYTRLGGMVWRADTKSNVYGKNHDTGVSPVFAGGVEYAITPEIATRLEYQWTNNIGDAHTI**GTRPD**NGMLSLGVSYRFGQGEAAPVVAPAPAPAPEVQTKHFTLKSDVLFNFNKATLKPEGQAALDQLYSQLSNLDPKDGSVVVLGYTDRIGSDAYNQGLSERRAQSVVDYLISKGIPADKISARGMGESNPVTGNTCDNVKQRAALIDCLAPDRRVEIEVKGIKDVVTQPQA

**Pnp**

**>gi|1172545|sp|P05055|PNP_ECOLI Polyribonucleotide nucleotidyltransferase (Polynucleotide phosphorylase) (PNPase)**

MLNPIVRKFQYGQHTVTLETGMMARQATAAVMVSMDDTAVFVTVVGQKKAKPGQDFFPLTVNYQERTYAAGRIPGSFFRREGRPSEGETLIARLIDRPIRPLFPEGFVNEVQVIATVVSVNPQVNPDIVAMIGASAALSLSGIPFNGPIGAARVGYINDQYVLNPTQDELKESKLDLVVAGTEAAVLMVESEAQLLSEDQMLGAVVFGHEQQQVVIQNINELVKEAGKPRWDWQPEPVNEALNARVAALAEARLSDAYRITDKQERYAQVDVIKSETIATLLAEDETLDENELGEILHAIEKNVVRSRVLAGEPRIDGREKDMIRGLDVRTGVLPRTHGSALFTRGETQALVTATLGTARDAQVLDELMGERTDTFLFHYNFPPYSVGETGMVG**SPKRR**EIGHGRLAKRGVLAVMPDMDKFPYTVRVVSEITESNGSSSMASVCGASLALMDAGVPIKAAVAGIAMGLVKEGDNYVVLSDILGDEDHLGDMDFKVAGSRDGISALQMDIKIEGITKEIMQVALNQAKGARLHILGVMEQAINAPRGDISEFAPRIHTIKINPDKIKDVIGKGGSVIRALTEETGTTIEIEDDGTVKIAATDGEKAKHAIRRIEEITAEIEVGRVYTGKVTRIVDFGAFVAIGGGKEGLVHISQIADKRVEKVTDYLQMGQEVPVKVLEVDRQGRIRLSIKEATEQSQPAAAPEAPAAEQGE

**ProS**

**gi|34395980|sp|P16659|SYP_ECOLI Prolyl-tRNA synthetase (Proline--tRNA ligase) (ProRS) (Global RNA synthesis factor)**

MRTSQYLLSTLKETPADAEVISHQLMLRAGMIRKLASGLYTWLPTGVRVLKKVENIVREEMNNAGAIEVSMPVVQPADLWQESGRWEQYGPELLRFVDRGERPFVLGPTHEEVITDLIRNELSSYKQLPLNFYQIQTKFRDEVRPRFGVMRSREFLMKDAYSFHTSQESLQETYDAMYAAYSKIFSRMGLDFRAVQADTGSIGGSASHEFQVLAQSGEDDVVFSDTSDYAANIELAEAIAPKEPRAAATQEMTLVDTPNAKTIAELVEQFNLPIEKTVKTLLVKAVEGSSFPQVALLVRGDHELNEVKAEKLPQVASPLTFATEEEIRAVVKAGPGSLGPVNMPIPVVIDRTVAAMSDFAAGANIDGKHYFGINWDRDVATPEVADIRNVVAGDPSPDGQGRLLIKRGIEVGHIFQLGTKYSEALKASVQGEDGRNQILTMGCYGIGVTRVVAAAIEQNYDERGIVWPDAIAPFQVAILPMNMHKSFRVQELAEKLYSELRAQGIEVLLDDRKERPGVMFADMELIGIPHTIVLGDRNLDNDDIEYKYRRNGEKQLIKTGDIVEYLVKQIKG

**PurA**

**>gi|67471590|sp|P0A7D4|PURA_ECOLI Adenylosuccinate synthetase (IMP--aspartate ligase) (AdSS) (AMPSase)**

MGNNVVVLGTQWGDEGKGKIVDLLTERAKYVVRYQGGHNAGHTLVINGEKTVLHLIPSGILRENVTSIIGNGVVLSPAALMKEMKELEDRGIPVRERLLLSEACPLILDYHVALDNAREKARGAKAIGTTGRGIGPAYEDKVARRGLRVGDLFDKETFAEKLKEVMEYHNFQLVNYYKAEAVDYQKVLDDTMAVADILTSMVVDVSDLLDQARQRGDFVMFEGAQGTLLDIDHGTYPYVTSSNTTAGGVATGSGLGPRYVDYVLGILKAYSTRVGAG**PFPT**ELFDETGEFLCKQGNEFGATTGRRRRTGWLDTVAVRRAVQLNSLSGFCLTKLDVLDGLKEVKLCVAYRMPDGREV**TTTP**LAADDWKGVEPIYETMPGWSESTFGVKDRSGLPQAALNYIKRIEELTGVPIDIISTG**PDRT**ETMILRDPFDA

**RpoB**

**>gi|67472412|sp|P0A8V2|RPOB_ECOLI DNA-directed RNA polymerase beta chain (RNAP beta subunit) (Transcriptase beta chain) (RNA polymerase beta subunit)**

MVYSYTEKKRIRKDFGKRPQVLDVPYLLSIQLDSFQKFIEQDPEGQYGLEAAFRSVFPIQSYSGNSELQYVSYRLGEPVFDVQECQIRGVTYSAPLRVKLRLVIYEREAPEGTVKDIKEQEVYMGEIPLMTDNGTFVINGTERVIVSQLHRSPGVFFDSDKGKTHSSGKVLYNARIIPYRGSWLDFEFDPKDNLFVRIDRRRKLPATIILRALNYTTEQILDLFFEKVIFEIRDNKLQMELVPERLRGETASFDIEANGKVYVEKGRRITARHIRQLEKDDVKLIEVPVEYIAGKVVAKDYIDESTGELICAANMELSLDLLAKLSQSGHKRIETLFTNDLDHGPYISETLRVDPTNDRLSALVEIYRMMRPGEPPTREAAESLFENLFFSEDRYDLSAVGRMKFNRSLLREEIEGSGILSKDDIIDVMKKLIDIRNGKGEVDDIDHLGNRRIRSVGEMAENQFRVGLVRVERAVKERLSLGDLDTLMPQDMINAKPISAAVKEFFGSSQLSQFMDQNNPLSEITHKRRISALGPGGLTRERAGFEVRDVHPTHYGRVCPIETPEGPNIGLINSLSVYAQTNEYGFLE**TPYRK**VTDGVVTDEIHYLSAIEEGNYVIAQANSNLDEEGHFVEDLVTCRSKGESSLFSRDQVDYMDVSTQQVVSVGASLIPFLEHDDANRALMGANMQRQAVPTLRADKPLVGTGMERAVAVDSGVTAVAKRGGVVQYVDASRIVIKVNEDEMYPGEAGIDIYNLTKYTRSNQNTCINQMPCVSLGEPVERGDVLADGPSTDLGELALGQNMRVAFMPWNGYNFEDSILVSERVVQEDRFTTIHIQELACVSRDTKLGPEEITADIPNVGEAALSKLDESGIVYIGAEVTGGDILVGKVTPKGETQLTPEEKLLRAIFGEKASDVKDSSLRVPNGVSGTVIDVQVFTRDGVEKDKRALEIEEMQLKQAKKDLSEELQILEAGLFSRIRAVLVAGGVEAEKLDKLPRDRWLELGLTDEEKQNQLEQLAEQYDELKHEFEKKLEAKRRKITQGDDLAPGVLKIVKVYLAVKRRIQPGDKMAGRHGNKGVISKINPIEDMPYDENGTPVDIVLNPLGVPSRMNIGQILETHLGMAAKGIGDKINAMLKQQQEVAKLREFIQRAYDLGADVRQKVDLSTFSDEEVMRLAENLRKGMPIATPVFDGAKEAEIKELLKLGDLPTSGQIRLYDGRTGEQFE**RPVT**VGYMYMLKLNHLVDDKMHARSTGSYSLVTQQPLGGKAQFGGQRFGEMEVWALEAYGAAYTLQEMLTVKSDDVNGRTKMYKNIVDGNHQMEPGMPESFNVLLKEIRSLGINIELEDE

**RpsA**

**>gi|30062446|ref|NP_836617.1| 30S ribosomal protein S1 [Shigella flexneri 2a str. 2457T]**

MTESFAQLFEESLKEIETRPGSIVRGVVVAIDKDVVLVDAGLKSESAIPAEQFKNAQGELEIQVGDEVDVALDAVEDGFGETLLSREKAKRHEAWITLEKAYEDAETVTGVINGKVKGGFTVELNGIRAFLPGSLVDVRPVRDTLHLEGKELEFKVIKLDQKRNNVVVSRRAVIESENSAERDQLLENLQEGMEVKGIVKNLTDYGAFVDLGGVDGLLHITDMAWKRVKHPSEIVNVGDEITVKVLKFDRERTRVSLGLKQLGEDPWVAIA**KRYP**EGTKLTGRVTNLTDYGCFVEIEEGVEGLVHVSEMDWTNKNIHPSKVVNVGDVVEVMVLDIDEERRRISLGLKQCKANPWQQFAETHNKGDRVEGKIKSITDFGIFIGLDGGIDGLVHLSDISWNVAGEEAVREYKKGDEIAAVVLQVDAERERISLGVKQLAEDPFNNWVALNKKGAIVTGKVTAVDAKGATVELADGVEGYLRASEASRDRVEDATLVLSVGDEVEAKFTGVDRKNRAISLSVRAKDEADEKDAIATVNKQEDANFSNNAMAEAFKAAKGE

**SucC**

**>gi|67473117|sp|P0A836|SUCC_ECOLI Succinyl-CoA synthetase beta chain (SCS-beta)**

MNLHEYQAKQLFARYGLPAPVGYA**CTTPRE**AEEAASKIGAGPWVVKCQVHAGGRGKAGGVKVVNSKEDIRAFAENWLGKRLVTYQTDANGQPVNQILVEAATDIAKELYLGAVVDRSSRRVVFMASTEGGVEIEKVAEETPHLIHKVALDPLTGPMPYQGRELAFKLGLEGKLVQQFTKIFMGLATIFLERDLALIEINPLVITKQGDLICLDGKLGADGNALFRQPDLREMRDQSQEDPREAQAAQWELNYVALDGNIGCMVNGAGLAMGTMDIVKLHGGEPANFLDVGGGATKERVTEAFKIILSDDKVKAVLVNIFGGIVRCDLIADGIIGAVAEVGVNVPVVVRLEGNNAELGAKKLADSGLNIIAAKGLTDAAQQVVAAVEGK

**Tsf**

**>gi|67462328|sp|P0A6P1|EFTS_ECOLI Elongation factor Ts (EF-Ts)**

MAEITASLVKELRERTGAGMMDCKKALTEANGDIELAIENMRKSGAIKAAKKAGNVAADGVIKTKIDGNYGIILEVNCQTDFVAKDAGFQAFADKVLDAAVAGKITDVEVLKAQFEEERVALVAKIGENINIRRVAALEGDVLGSYQHGARIGVLVAAKGADEELVKHIAMHVAASKPEFIKPEDVSAEVVEKEYQVQLDIAMQSGKPKEIAEKMVEGRMKKFTGEVSLTGQPFVME**PSKT**VGQLLKEHNAEVTGFIRFEVGEGIEKVETDFAAEVAAMSKQS

**TufB**

**>gi|30064737|ref|NP_838908.1: 394 protein chain elongation factor EF-Tu [Shigella flexneri 2a str. 2457T]**

MSKEKFERTKPHVNVGTIGHVDHGKTTLTAAITTVLAKTYGGAARAFDQIDNAPEEKARGITINTSHVEYDTPTRHYAHVDCPGHADYVKNMITGAAQMDGAILVVAATDGPMPQTREHILLGRQVGVPYIIVFLNKCDMVDDEELLELVEMEVRELLSQYDFPGDDTPIVRGSALKALEGDAEWEAKILELAGFLDSYIPEPERAIDKPFLLPIEDVFSISGRGTVVTGRVERGIIKVGEEVEIVGIKETQKSTCTGVEMFRKLLDEGRAGENVGVLLRGIKREEIERGQVLAKPG**TIKPHTK**FESEVYILSKDEGG**RHTP**FFKGYRPQFYFRTTDVTGTIELPEGVEMVMPGDNIKMVVTLIHPIAMDDGLRFAIREGGRTVGAGVVAKVLS

**YaeT**

**>gi|71164818|sp|P0A940|YAET_ECOLI Outer membrane protein assembly factor yaeT precursor (Omp85)**

MAMKKLLIASLLFSSATVYGAEGFVVKDIHFEGLQRVAVGAALLSMPVRTGDTVNDEDISNTIRALFATGNFEDVRVLRDGDTLLVQVKERPTIASITFSGNKSVKDDMLKQNLEASGVRVGESLDRTTIADIEKGLEDFYYSVGKYSASVKAVVTPLPRNRVDLKLVFQEGVSAEIQQINIVGNHAFTTDELISHFQLRDEVPWWNVVGDRKYQKQKLAGDLETLRSYYLDRGYARFNIDSTQVSL**TPDKK**GIYVTVNITEGDQYKLSGVEVSGNLAGHSAEIEQLTKIEPGELYNGTKVTKMEDDIKKLLGRYGYAYPRVQSMPEINDADKTVKLRVNVDAGNRFYVRKIRFEGNDTSKDAVLRREMRQMEGAWLGSDLVDQGKERLNRLGFFETVDTDTQRVPGSPDQVDVVYKVKERNTGSFNFGIGYGTESGVSFQAGVQQDNWLGTGYAVGINGTKNDYQTYAELSVTNPYFTVDGVSLGGRLFYNDFQADDADLSDYTNKSYGTDVTLGFPINEYNSLRAGLGYVHNSLSNMQPQVAMWRYLYSMGEHPSTSDQDNSFKTDDFTFNYGWTYNKLDRGYFPTDGSRVNLTGKVTIPGSDNEYYKVTLDTATYVPIDDDHKWVVLGRTRWGYGDGLGGKEMPFYENFYAGGSSTVRGFQSNTIGPKAVYFPHQASNYDPDYDYECATQDGAKDLCKSDDAVGGNAMAVASLEFITPTPFISDKYANSVRTSFFWDMGTVWDTNWDSSQYSGYPDYSDPSNIRMSAGIALQWMSPLGPLVFSYAQPFKKYDGDKAEQFQFNIGKTW
