# Supplementary material for: Rules Governing Selective Protein Carbonylation
Source: PLoS One. 2009 Oct 5;4(10):e7269. doi: 10.1371/journal.pone.0007269 (PMC2751825; doi:10.1371/journal.pone.0007269)
Supplement: Table S1 — Parameters for the identification of RKPT-enriched regions The primary sequence of BSA contains an average of one carbonylatable site within a sequence of 4 amino acids (23.5% of carbonylatable sites); thus, we tested how to define an RKPT-enriched region by analysing the specificity, the sensitivity and the positive predictive value of four enrichments, from 1 to 4 carbonylatable sites, within a sequence of 4 amino acids. (0.03 MB DOC) [file pone.0007269.s006.doc]

| **Defined RKPT enriched region (Number of carbonylatable sites within a window of four residues)** | | | | | | | | |
| --- | --- | --- | --- | --- | --- | --- | --- | --- |
|  | **1/4** | | **2/4** | | **3/4** | | **4/4** | |
| **Residues** | **carbonylated** | **Uncarbonylated** | **carbonylated** | **uncarbonylated** | **carbonylated** | **uncarbonylated** | **carbonylated** | **uncarbonylated** |
| **inside RKPT enriched regions** | 26 | 107 | 25 | 76 | 17 | 27 | 0 | 0 |
| **outside RKPT enriched regions** | 0 | 0 | 1 | 31 | 9 | 80 | 26 | 107 |
| **Sensitivity** | 1 | | 0.96 | | 0.65 | | 0 | |
| **Specificity** | 0 | | 0.29 | | 0.75 | | 1 | |
| **Positive predictive value** | 0.19 | | 0.25 | | 0.39 | | nd | |
