# Supplementary material for: Rules Governing Selective Protein Carbonylation
Source: PLoS One. 2009 Oct 5;4(10):e7269. doi: 10.1371/journal.pone.0007269 (PMC2751825; doi:10.1371/journal.pone.0007269)
Supplement: Table S2 — MS data for MALDI-TOF global mass analysis of BSA (*) M/Z of BSA monomer were obtained with standard deviations of 1000 ppm (▒) As the ionisation energy was the same for each sample, the half height width of the BSA monomer accounted for the BSA molecule heterogeneity. However, due to the quantitative decrease of BSA monomer (for instance at MCO level 1), the signal to noise ratio decreased leading to an over-estimation of the height width of the BSA monomer. (0.03 MB DOC) [file pone.0007269.s007.doc]

| **Oxidation levels** | **M/Z monomer of BSA (Da)*** | **Width of half height of BSA monomer*$** |
| --- | --- | --- |
| 0 | 66431 | 624 |
| 10^-3^ | 66492 | 721 |
| 10^-2^ | 66505 | 757 |
| 10^-1^ | 66523 | 895 |
| 1 | 66989 | 1070 |
