# Supplementary material for: Rules Governing Selective Protein Carbonylation
Source: PLoS One. 2009 Oct 5;4(10):e7269. doi: 10.1371/journal.pone.0007269 (PMC2751825; doi:10.1371/journal.pone.0007269)
Supplement: Table S3 — Data analysis of CS found in the proteins analysed by nano-LC ESI MS/MS. List of all carbonylated peptides containing at least one CS as judged by mass spectrometry analysis. CS and oxidised methionines are shaded in gray. Whenever possible, assessment of the structural environment and of the solvent exposure of isolated CS was done by visual inspection of the relevant pdb or homologue files. CS were thus mapped within (1) apha helices, (2) loops, and (3) beta strands. Asterisks (*) show CS not conserved in the corresponding PDB homologue. For those cases where structural data were either completely ▒ (NP_415431.1, NP_414719.1) or partially lacking (NP_414555.1, NP_417799.1, NP_414596.1, NP_415477.1 and NP_418414.1), we carried out structural predictions to locate residues in ordered or disordered (see experimental procedures). (0.08 MB DOC) [file pone.0007269.s008.doc]

| **Gene name** | **Accession number** | **PDB ID** | **Coverage** | **Carbonylatable sites** | | | | | **CS** | | | | | **Location of CS** | | | | **Identified carbonylated peptide** |
| --- | --- | --- | --- | --- | --- | --- | --- | --- | --- | --- | --- | --- | --- | --- | --- | --- | --- | --- |
|  |  |  |  | **P** | **K** | **R** | **T** | **Total** | **P** | **K** | **R** | **T** | **Total** | **Surface** | **Disordered region** | **Buried** | **Unknown** |  |
| *aceE* | NP_414656.1 | 2G28 | 59% | 23 | 30 | 25 | 28 | 106 | 1 | - | - | - | 1 | ^(1)^P_787_ |  |  |  | R_(785)_.VPYIAQVMNDAPAVASTDYMK.L_(807)_ |
| *acnB* | NP_414660.1 | 1L5J | 51% | 28 | 21 | 17 | 31 | 97 | 3 | 1 | 1 | - | 5 | ^(1)^K_387_ ^(1)^R_391_ ^(2)^P_489_ |  | ^(2)^P_739_ ^(2)^P_740_ |  | R_(735)_.LWAPPTRMDAAQLTEEGYYSVFGK.S_(761)_  K_(373)_.DVAESDRGFSLAQKMVGR.A_(392)_  R_(485)_.MLLPDTVGTGGDSHTR.F_(502)_ |
| *atpD* | NP_418188.1 | 2JDI  (E-F) | 91% | 19 | 12 | 22 | 21 | 74 | 3 | - | - | - | 3 | ^(2)^P_75_ ^(3)^P_79_ ^(2)^P_115_ |  |  |  | K_(70)_.DLEHPIEVPVGK.A_(83)_ or K_(70)_.DLEHPIEVPVGK.A_(83)_  K_(70)_.DLEHPIEVPVGK.A_(83)_  R_(112)_.AAPSYEELSNSQELLETGIK.V_(133)_ |
| *clpB* | NP_417083.1 | 1QVR | 34% | 14 | 8 | 15 | 12 | 49 | - | - | 1 | - | 1 | ^*(1)^R_418_ |  |  |  | R_(417)_.RIIQLK.L_(424)_ |
| *dnaK* | NP_414555.1 | 1DKG  (D) | 66% | 15 | 24 | 29 | 20 | 88 | 2 | - | 1 | 4 | 7 | ^(1)^R_56_ ^(1)^T_60_ ^(2)^P_90_ | T_416_ T_417_  P_419_ P_420_ |  |  | K_(414)_.NTTIPTKHSQVFSTAEDNQSAVTIHVLQGER.K_(446)_  K_(55)_.RQAVTNPQNTLFAIK.R_(71)_  R_(76)_.FQDEEVQRDVSIMPFK.I_(93)_ |
| *eno* | NP_417259.1 | 2FYM  (A-D) | 62% | 6 | 17 | 3 | 12 | 38 | 1 | - | - | 1 | 2 | ^(2)^T_204_ ^(2)^P_214_ |  |  |  | K_(201)_.GMNTAVGDEGGYAPNLGSNAEALAVIAEAVK.A_(233)_ |
| *fabH* | NP_415609.1 | 2GYO (A,B) | 34% | 4 | 1 | 6 | 9 | 20 | 1 | - | - | - | 1 | ^(2)^P_47_ |  |  |  | R_(42)_.HIAAPNETVSTMGFEAATR.A_(62)_ |
| *fhuA* | NP_414692.1 | 1BY5  (A) | 40% | 17 | 13 | 11 | 21 | 62 | 1 | - | - | - | 1 | ^(2)^P_96_ |  |  |  | K_(78)_.VPQSISVVTAEEMALHQPK.S_(98)_ |
| *fusA* | NP_417799.1 | 1KTV (A,B) | 63% | 26 | 24 | 21 | 20 | 91 | 3 | 1 | - | 2 | 6 | ^*(3)^T_348_ ^(3)^P_524_ ^(2)^P_570_ ^(2)^K_609_ ^*(2)^T_681_ |  | ^(3)^P_615_ |  | R_(337)_.VYSGVVNSGDTVLNSVKAAR.E_(358)_  R_(512)_.GQYGHVVIDMYPLEPGSNPK.G_(533)_  K_(562)_.AGPLAGYPVVDMGIR.L_(578)_  R_(677)_.ASYTMEFLKYDEAPSNVAQAVIEAR.G_(702)_  K_(605)_.KAKPVLLEPIMK.V_(619)_ |
| *groL* | NP_418567.1 | 2EU1  (A-N) | 80% | 12 | 32 | 17 | 29 | 90 | 4 | 2 | 2 | 3 | 11 | ^(2)^T_30_ ^(2)^P_3_ ^(2)^K_34_ ^(1)^P_496_ ^(1)^T_497_ ^(1)^T_500_ ^(1)^R_501_ ^(1)^K_498_ |  | ^(2)^R_36_, ^(1)^P_113_, ^(1)^P_235_ |  | K_(468)_.GGDGNYGYNAATEEYGNMIDMGILDPTK.V_(499)_  K_(468)_.GGDGNYGYNAATEEYGNMIDMGILDPTKVTR.S_(502)_  K_(468)_.GGDGNYGYNAATEEYGNMIDMGILDPTKVTR.S_(502)_  K_(468)_.GGDGNYGYNAATEEYGNMIDMGILDPTKVTR.S_(502)_  K_(28)_.VTLGPK.G_(35)_  K_(28)_.VTLGPKGRNVVLDK.S_(43)_  K_(104)_.AVAAGMNPMDLKRGIDK.A_(122)_  K_(79)_.ANDAAGDGTTTATVLAQAIITEGLKAVAAGMNPMDLKR.G_(118)_  K_(226)_.ISNIREMLPVLEAVAK.A_(243)_ |
| *htpG* | NP_415006.1 | 2IOP  (A-D) | 58% | 16 | 46 | 37 | 34 | 133 | 1 | - | 1 | 2 | 4 | ^(2)^T_535_ ^(2)^P_536_ ^(2)^T_541_ |  | ^(1)^R_33_ |  | R_(531)_.LTDTPAIVSTDADEMSTQMAK.L_(553)_  K_(28)_.EIFLRELISNASDAADK.L_(46)_ |
| *imp* | NP_414596.1 | 1KV9  (A) | 49% | 13 | 10 | 21 | 22 | 66 | - | - | 1 | - | 1 |  |  |  | R_650_ | R_(645)_.RDEDRLVQLNYR.Y_(658)_ |
| *leuS* | NP_415175.1 | 2V0G  (A, D) | 30 | 9 | 17 | 16 | 11 | 53 | - | - | - | 1 | 1 | ^(1)^T_695_ |  |  |  | K_(683)_.GDVAALNVDALTENQK.A_(700)_ |
| *ompA* | NP_415477.1 | 2GE4  (A) | 73% | 15 | 15 | 11 | 17 | 58 | 4 | 1 | - | 3 | 8 |  | P_198_ P_204_  P_206_ P_208_ T_212_ K_213_ T_216_ T_127_ |  |  | R_(190)_.FGQGEAAPVVAPAPAPAPEVQTKHFTLK.S_(219)_  R_(190)_.FGQGEAAPVVAPAPAPAPEVQTKHFTLK.S_(219)_  R_(190)_.FGQGEAAPVVAPAPAPAPEVQTKHFTLK.S_(219)_  R_(190)_.FGQGEAAPVVAPAPAPAPEVQTKHFTLK.S_(219)_  R_(190)_.FGQGEAAPVVAPAPAPAPEVQTK.H_(214)_  R_(124)/_ADTKSNVYGK.N_(135)_ |
| *pnp* | NP_417633.3 | 1E3P | 51% | 13 | 19 | 12 | 22 | 66 | 1 | - | - | - | 1 | ^*(2)^P_544_ |  |  |  | R_(530)_.LHILGVMEQAINAPR.G_(546_) |
| *proS* | NP_414736.1 | 2J3M  (A,B) | 48% | 17 | 13 | 14 | 18 | 62 | 1 | - | - | - | 1 | ^*(2)^P_340_ |  |  |  | K_(332)_.AGPGSLGPVNMPIPVVIDR.T_(352)_ |
| *purA* | NP_418598.1 | 1KKF  (A) | 45% | 9 | 12 | 8 | 16 | 45 | 3 | - | 1 | 3 | 7 | ^(2)^P_410_ ^(2)^P_418_ ^(2)^R_420_ ^(2)^T_421_ ^(3)^T_423_ |  | ^(2)^T_416_, ^(2)^P_429_ |  | K_(401)_.RIEELTGVPDIISTGPDRTETMILR.D_(427)_  K_(401)_.RIEELTGVPDIISTGPDRTETMILRDPFDA.-_(432)_ |
| *rpoB* | NP_418414.1 | 2PPB  (C, M) | 31% | 12 | 24 | 33 | 11 | 80 | 1 | 1 | 2 | - | 4 | ^*(1)^R_1301_ |  |  | K_203_ P_993_ R_994_ | R_(976)_.AVLVAGGVEAEKLDKLPR.D_(995)_  R_(200)_.RKLPATIILR.A_(212)_  [K_(1294)_.SDDVNGRTKMYK.N](mailto:K.SDDVNGR@TKM*YK.N)_(1307)_ |
| *rpsA* | NP_415431.1 | 1EFU  (B, D) | 66% | 9 | 28 | 22 | 16 | 75 | - | - | - | 1 |  |  |  |  | T_270_ | R_(273)_.YPEGTKLTGR.V_(284)_ |
| *sucC* | NP_415256.1 | 1JKJ  (B, E) | 56% | 5 | 12 | 8 | 8 | 33 | 1 | - | 1 | - | 2 | ^(1)^P_227_ ^(1)^R_230_ |  |  |  | R_(225)_.QPDLREMR.D_(234)_ |
| *tsf* | NP_414712.1 |  | 66% | 3 | 15 | 6 | 7 | 31 | - | - | - | 1 | 1 | ^(2)^T_270_ |  |  |  | R_(258)_.FEVGEGIEKVETDFAAEVAAMSK.Q_(282)_ |
| *tufB* | NP_417798.1 | 2FX3  (A) | 78% | 17 | 19 | 17 | 26 | 79 | 7 | 1 | 1 | 4 | 13 | ^(2)^P_210_ ^(3)^P_214_ ^(2)^P_296_ ^(2)^T_298_ ^(3)^P_301_ ^(3)^T_303_ ^(2)^R_319_ ^(2)^T_321_ ^(2)^P_322_  ^(2)^P_353_ ^(3)^T_383_ ^3)^K_391_ |  | ^(3)^P_366_ |  | K_(295)_.PGTIKPHTKFESEVYILSK.D_(315)_  K_(295)_.PGTIKPHTKFESEVYILSK.D_(315)_  K_(295)_.PGTIKPHTKFESEVYILSK.D_(315)_  K_(314)_.DEGGRHTPFFK._(326)_  R_(205)_.AIDKPFLLPIEDVFSISGR.G_(225)_  R_(205)_.AIDKPFLLPIEDVFSISGR.G_(225)_  R_(373)_.FAIREGGRTVGAGVVAKVLS.-_(386)_  R_(334)_.TTDVTGTIELPEGVEMVMPGDNIK.M_(359)_ K_(358)_.MVVTLIHPIAMDDGLR.F_(375)_ |
| *^$^yaeT* | NP_414719.1 |  | 50% | 14 | 24 | 23 | 28 | 89 | 1 | - | - | - | 1 |  | P_326_ |  |  | R_(321)_.VQSMPEINDADK.T_(334)_ |
| total |  |  |  | 316 | 436 | 394 | 439 | 1585 | 39 | 7 | 12 |  | 83 | 55 | 13 | 9 | 6 |  |
