# Supplementary material for: Rules Governing Selective Protein Carbonylation
Source: PLoS One. 2009 Oct 5;4(10):e7269. doi: 10.1371/journal.pone.0007269 (PMC2751825; doi:10.1371/journal.pone.0007269)
Supplement: Table S4 — Set of E. coli proteins specifically carbonylated or uncarbonylated. The table shows three sets of proteins used to test the efficiency of the CSPD model. CP from exponentially grown cells were provided from this study. CP from the stationary phase were obtained from several studies already carried out on E. coli [1], [2]. Non-CP were provided from this study. Proteins containing at least one predicted HSC are indicated by an asterisk. [1] Dukan S, Nystrom T (1998) Bacterial senescence: stasis results in increased and differential oxidation of cytoplasmic proteins leading to developmental induction of the heat shock regulon. Genes Dev 12: 3431–3441. [2] Dukan S, Nystrom T (1999) Oxidative stress defense and deterioration of growth-arrested Escherichia coli cells. J Biol Chem 274: 26027-26032. (0.07 MB DOC) [file pone.0007269.s009.doc]

| **CP from exponentially grown *E. coli*** | | | **CP from stationary grown *E. coli*** | | | **Uncarbonylated protein from exponentially grown *E. coli*** | | |
| --- | --- | --- | --- | --- | --- | --- | --- | --- |
| **Gene name** | **Accession number** | **Functional description** | **Gene name** | **Accession number** | **Functional description** | **Gene name** | **Accession number** | **Functional description** |
| *aceE** | NP_414656.1 | pyruvate dehydrogenase subunit E1 | *aceF* | NP_414657.1 | dihydrolipoamide acetyltransferase | ftsZ | NP_414637.1 | cell division protein |
| *acnB** | NP_414660.1 | aconitate hydratase | *carA** | NP_414573.1 | carbamoyl-phosphate synthase small subunit | dapD | NP_414708.1 | 2,3,4,5-tetrahydropyridine-2-carboxylate |
| *atpD* | NP_418188.1 | F0F1 ATP synthase subunit beta | *fabB** | NP_416826.1 | 3-oxoacyl-(acyl carrier protein) synthase | tsx | NP_414945.1 | nucleoside channel, receptor of phage T6 |
| *clpB** | NP_417083.1 | protein disaggregation chaperone | *glnA** | NP_418306.1 | glutamine synthetase | lon | NP_414973.1 | DNA-binding ATP-dependent protease |
| *dnaK** | NP_414555.1 | molecular chaperone DnaK | *gltD* | NP_417680.1 | glutamate synthase, 4Fe-4S protein, | acrA | NP_414996.1 | multidrug efflux system |
| *eno** | NP_417259.1 | phosphopyruvate hydratase | *glyA* | NP_417046.1 | serine hydroxymethyltransferase | ahpC | NP_415138.1 | alkyl hydroperoxide reductase, C22 subunit |
| *fabH* | NP_415609.1 | 3-oxoacyl-(acyl carrier protein) synthase | *gnd** | NP_416533.1 | 6-phosphogluconate dehydrogenase | asnB | NP_415200.1 | asparagine synthetase B |
| *fhuA** | NP_414692.1 | ferrichrome outer membrane transporter | *icd** | NP_415654.1 | isocitrate dehydrogenase | ybhC | NP_415293.1 | predicted pectinesterase |
| *fusA** | NP_417799.1 | elongation factor EF-2 | *mdh* | NP_417703.1 | malate dehydrogenase | fiu | NP_415326.1 | predicted iron outer membrane transporter |
| *groL** | NP_418567.1 | chaperonin GroEL | *metE** | NP_418273.1 | 5-methyltetrahydropteroyltriglutamate | fabI | NP_415804.1 | enoyl-(acyl carrier protein) reductase |
| *htpG* | NP_415006.1 | Heat shock protein 90 | *ptsI* | NP_416911.1 | PEP-protein phosphotransferase | gadB | NP_416010.1 | glutamate decarboxylase B, PLP-dependent |
| *imp** | NP_414596.1 | organic solvent tolerance protein | *purL** | YP_026170.1 | phosphoribosylformylglycinamidine synthase | gapA | NP_416293.1 | glyceraldehyde-3-phosphate dehydrogenase |
| *leuS** | NP_415175.1 | leucyl-tRNA synthetase | *pykA* | NP_416368.1 | pyruvate kinase | eda | NP_416364.1 | keto-hydroxyglutarate-aldolase |
| *ompA** | NP_415477.1 | outer membrane protein A (3a;II*;G;d) | *pykF** | NP_416191.1 | pyruvate kinase | rfbA | NP_416543.1 | glucose-1-phosphate thymidylyltransferase |
| *pnp** | NP_417633.3 | polynucleotide phosphorylase | *serA* | NP_417388.1 | D-3-phosphoglycerate dehydrogenase | rho | NP_418230.1 | transcription termination factor Rho |
| *proS* | |NP_414736.1 | prolyl-tRNA synthetase | *sucB** | NP_415255.1 | dihydrolipoamide acetyltransferase | udp | NP_418275.1 | uridine phosphorylase |
| *purA** | NP_418598.1 | adenylosuccinate synthetase | *znuA* | NP_416371.4 | high-affinity zinc transporter periplasmic | rplA | NP_418411.1 | 50S ribosomal protein L1 |
| *rpoB** | NP_418414.1 | DNA-directed RNA polymerase |  |  |  | lamB | NP_418460.1 | maltoporin precursor |
| *rpsA** | NP_415431.1 | 30S ribosomal protein S1 |  |  |  | ppa | NP_418647.1 | inorganic pyrophosphatase |
| *sucC** | NP_415256.1 | succinyl-CoA synthetase subunit beta |  |  |  | flu | YP_026164.1 | CP4-44 prophage; antigen 43 (Ag43) |
| *tsf** | NP_414712.1 | elongation factor Ts |  |  |  | sodA | NP_418344.3 | superoxide dismutase, Mn |
| *tufB** | NP_417798.1 | protein chain elongation factor EF-Tu |  |  |  | upp | NP_416993.2 | uracil phosphoribosyltransferase |
| *yaeT** | NP_414719.1 | hypothetical protein |  |  |  | galF | NP_416546.1 | predicted subunit with GalU |
|  |  |  |  |  |  | cirA | NP_416660.1 | ferric iron-catecholate outer membrane transporter |
|  |  |  |  |  |  | fadL | NP_416846.1 | long-chain fatty acid outer membrane transporter |
|  |  |  |  |  |  | infB | NP_417637.1 | translation initiation factor IF-2 |
|  |  |  |  |  |  | NusA | NP_417638.1 | transcription elongation factor |
|  |  |  |  |  |  | rplC | NP_417779.1 | 50S ribosomal protein L3 |
